# Differential and Overlapping Effects of 20,23(OH)_2_D3 and 1,25(OH)_2_D3 on Gene Expression in Human Epidermal Keratinocytes: Identification of AhR as an Alternative Receptor for 20,23(OH)_2_D3

**DOI:** 10.3390/ijms19103072

**Published:** 2018-10-08

**Authors:** Andrzej T. Slominski, Tae-Kang Kim, Zorica Janjetovic, Anna A. Brożyna, Michal A. Żmijewski, Hui Xu, Thomas R. Sutter, Robert C. Tuckey, Anton M. Jetten, David K. Crossman

**Affiliations:** 1 Department of Dermatology, University of Alabama at Birmingham, Birmingham, AL 35294, USA; tkim@uabmc.edu (T.-K.K.); zjanjetovic@uabmc.edu (Z.J.); huixu@uabmc.edu (H.X.); 2 Comprehensive Cancer Center, Cancer Chemoprevention Program, University of Alabama at Birmingham, Birmingham, AL 35294, USA; 3 Veteran Administration Medical Center, Birmingham, AL 35294, USA; 4 Department of Medical Biology, Faculty of Biology and Environment Protection, Nicolaus Copernicus University, 87-100 Toruń, Poland; brozyna.anna@gmail.com; 5 Department of Tumor Pathology and Pathomorphology, Oncology Centre-Prof. Franciszek Łukaszczyk Memorial Hospital, 85-796 Bydgoszcz, Poland; 6 Department of Histology, Medical University of Gdańsk, 80-211 Gdańsk, Poland; mzmijewski@gumed.edu.pl; 7 Feinstone Center for Genomic Research, University of Memphis, Memphis, TN 38152 USA; tsutter@memphis.edu; 8 School of Molecular Sciences, The University of Western Australia, Perth, WA 6009, Australia; robert.tuckey@uwa.edu.au; 9 Immunity, Inflammation, and Disease Laboratory/Cell Biology Group, National Institute of Environmental Health Sciences, NIH, Research Triangle Park, NC 27709, USA; jetten@niehs.nih.gov; 10 Howell and Elizabeth Heflin Center for Human Genetics, Genomic Core Facility, University of Alabama at Birmingham, Birmingham, AL 35294, USA; dcrossman@uabmc.edu

**Keywords:** vitamin D, dihydroxyvitamin D, epidermal keratinocytes, nuclear receptor signaling, microarray

## Abstract

A novel pathway of vitamin D activation by CYP11A has previously been elucidated. To define the mechanism of action of its major dihydroxy-products, we tested the divergence and overlap between the gene expression profiles of human epidermal keratinocytes treated with either CYP11A1-derived 20,23(OH)_2_D3 or classical 1,25(OH)_2_D3. Both secosteroids have significant chemical similarity with the only differences being the positions of the hydroxyl groups. mRNA was isolated and examined by microarray analysis using Illumina’s HumanWG-6 chip/arrays and subsequent bioinformatics analyses. Marked differences in the up- and downregulated genes were observed between 1,25(OH)_2_D3- and 20,23(OH)_2_D3-treated cells. Hierarchical clustering identified both distinct, opposite and common (overlapping) gene expression patterns. *CYP24A1* was a common gene strongly activated by both compounds, a finding confirmed by qPCR. Ingenuity pathway analysis identified VDR/RXR signaling as the top canonical pathway induced by 1,25(OH)_2_D3. In contrast, the top canonical pathway induced by 20,23(OH)_2_D3 was AhR, with VDR/RXR being the second nuclear receptor signaling pathway identified. QPCR analyses validated the former finding by revealing that 20,23(OH)_2_D3 stimulated *CYP1A1* and *CYP1B1* gene expression, effects located downstream of AhR. Similar stimulation was observed with 20(OH)D3, the precursor to 20,23(OH)_2_D3, as well as with its downstream metabolite, 17,20,23(OH)_3_D3. Using a Human AhR Reporter Assay System we showed marked activation of AhR activity by 20,23(OH)_2_D3, with weaker stimulation by 20(OH)D3. Finally, molecular modeling using an AhR LBD model predicted vitamin D3 hydroxyderivatives to be good ligands for this receptor. Thus, our microarray, qPCR, functional studies and molecular modeling indicate that AhR is the major receptor target for 20,23(OH)_2_D3, opening an exciting area of investigation on the interaction of different vitamin D3-hydroxyderivatives with AhR and the subsequent downstream activation of signal transduction pathways in a cell-type-dependent manner.

## 1. Introduction

Vitamin D3 (D3) is formed by ultraviolet B radiation (UVB)-mediated breaking of the B ring of 7-dehydrocholesterol (7DHC) followed by thermal isomerization of the resulting pre-vitamin D3 to D3 [1,2]. The vast majority of circulating D3 is generated in epidermal keratinocytes [3]. D3 is a prohormone that is activated by sequential hydroxylations at C25 (by CYP2R1 or CYP27A1) and C1α (by CYP27B1) to 1,25-dihydroxyvitamin D3 (1,25(OH)_2_D3), the hormonally active form, referred to as the canonical pathway [4,5,6,7]. At the systemic level, C25 hydroxylation takes place in the liver with the resulting 25(OH)D3 being hydroxylated at C1α in kidneys [3,4,5,6,7]. The same pathway operates in peripheral tissues including epidermal keratinocytes, the major site of D3 formation [1]. 

In addition to the canonical pathway of vitamin D activation described above, novel CYP11A-mediated pathways have been discovered (reviewed in [8]). Specifically, CYP11A1, the first enzyme of steroidogenesis that hydroxylates and then cleaves the side chain of cholesterol to produce pregnenolone (reviewed in [9,10]), can also hydroxylate and cleave the side chain of 7DHC, and hydroxylate the side chain of D3 and D2 without cleavage [11,12,13,14,15,16]. The two major products of CYP11A1 action on D3, with defined stereochemistry, are 20*S*-hydroxyvitamin D3 (20(OH)D3) and 20*S*,23*S*-dihydroxyvitamin D3 (20,23(OH)_2_D3) [17]. These pathways operate in cultured epidermal, human and pig keratinocytes, dermal fibroblasts, colon cancer cells, and have also been described ex vivo for placenta and adrenal glands [18,19,20,21,22,23]. Importantly, the major products of these pathways are detectable in vivo in human serum, epidermis and adrenal glands [24].

The classical, hormonally-active dihydroxy form of vitamin D3, 1,25(OH)_2_D3, in addition to playing a fundamental role in body calcium and phosphorous homeostasis and in the proper functioning of the skeletomuscular system, has pleiotropic effects on different organs and cell functions (reviewed in [3,6,25,26,27,28,29]). These studies show that 1,25(OH)_2_D3 has immunomodulatory properties, is involved in the regulation of reproduction, pregnancy, child development, neurodevelopment, regulation of global metabolic and endocrine homeostasis and functions of the cardiovascular system, and has anticancer activities (reviewed in [2,6,30,31,32,33,34,35,36,37,38,39,40,41,42,43,44,45,46,47,48,49,50]). At the cellular level, it regulates proliferation, differentiation, apoptosis, senescence, metabolism, migration, secretory activities, and protective and reparative mechanisms against oxidative stress and radiation. It is widely accepted that these functions are regulated by different signal transduction pathways initiated by 1,25(OH)_2_D3 binding to the vitamin D receptor (VDR) at the genomic binding site, and to some degree at a nongenomic binding site, in a cell-type dependent manner (reviewed [6,45,46,47,51,52,53,54]). In the skin, 1,25(OH)_2_D3 regulates the epidermal barrier and hair cycling and has radioprotective, anti-cancer and anti-inflammatory properties [1,3,52,53,55,56,57,58,59]. 

The novel secosteroids, produced by the non-canonical activation pathways initiated by CYP11A1, inhibit the proliferation of epidermal keratinocytes, melanocytes and dermal fibroblasts and promote the differentiation of keratinocytes. Furthermore, they inhibit fibrotic activities of fibroblasts and have immunomodulatory properties (reviewed in [19,49,60]). Importantly, 20(OH)D3 and 20,23(OH)_2_D3 are non-calcemic at pharmacological doses [61,62,63] which is in contrast to the highly calcemic effects of 1,25(OH)_2_D3 and 25(OH)D3. 20(OH)D3 and 20,23(OH)_2_D3 also attenuate the symptoms of skin fibrosis, rheumatoid arthritis and have photoprotective properties [8,19,23,64,65]. The CYP11A1-derived secosteroids have pleiotropic phenotypic effects that are cell-type–dependent [19,23,60,61,62,65,66,67,68,69,70,71,72,73,74,75]. They can act as biased agonists of the VDR [19,60,76,77] and can act as inverse agonists on retinoic acid orphan receptors (ROR) α and γ [60,78]. 

To better define the signaling pathways and mechanisms underlying the similarities and differences between phenotypic activities of classical 1,25(OH)_2_D3 and the major dihydroxy product of CYP11A1 action on vitamin D3, 20,23(OH)_2_D3, we examined and compared the gene expression profiles of human keratinocytes exposed to these secosteroids. Bioinformatics analysis was performed and differences and similarities in the activities of these structurally similar but distinct dihydroxy-D3 species were compared.

## 2. Results and Discussion

**Figure 1:**
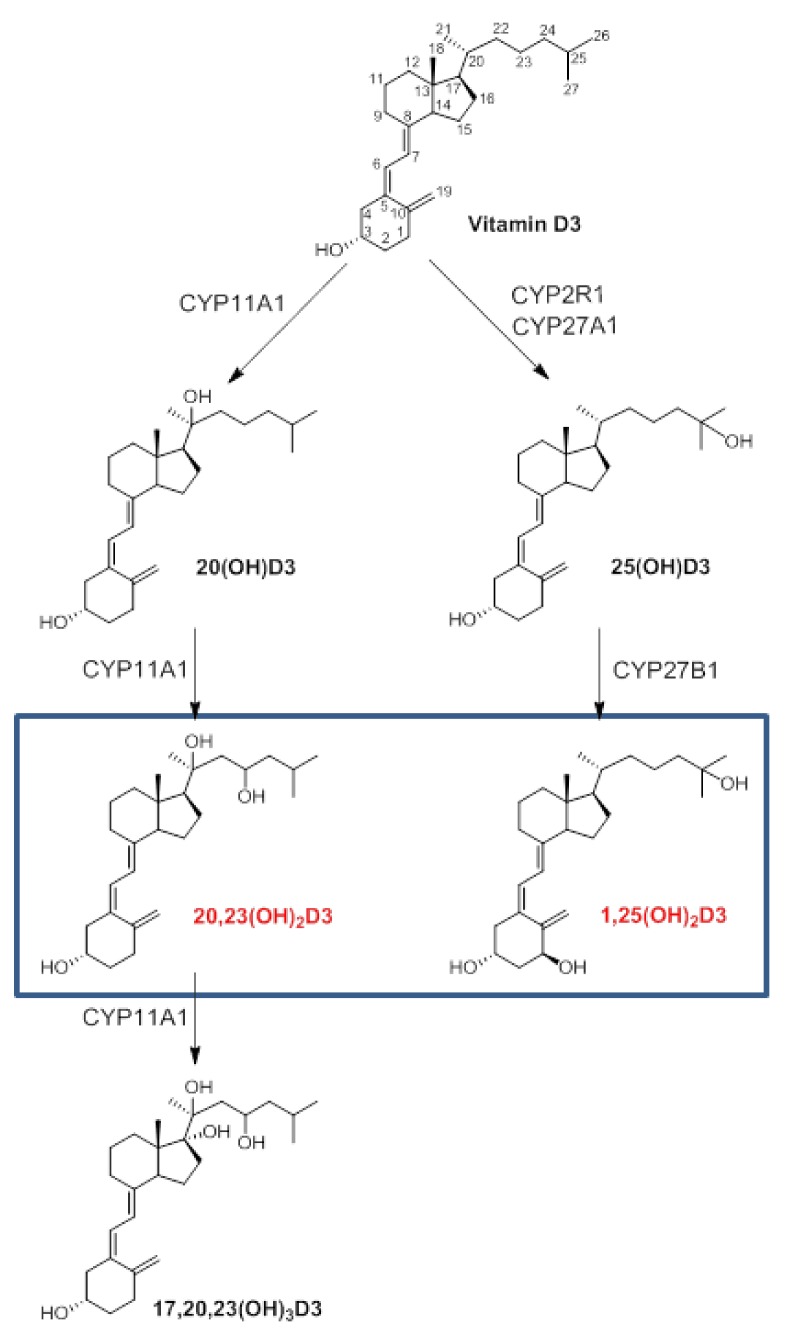
Epidermal pathways of vitamin D3 activation to produce 1,25(OH)_2_D3 and 20,23(OH)_2_D3 and the downstream metabolite 17,20,23(OH)_3_D3. The rectangle marks the secosteroids used for microarray analyses.

The structures and sequences of the reactions producing 1,25(OH)_2_D3 and 20,23(OH)_2_D3 in the epidermis are shown in Figure 1.

**Figure 2:**
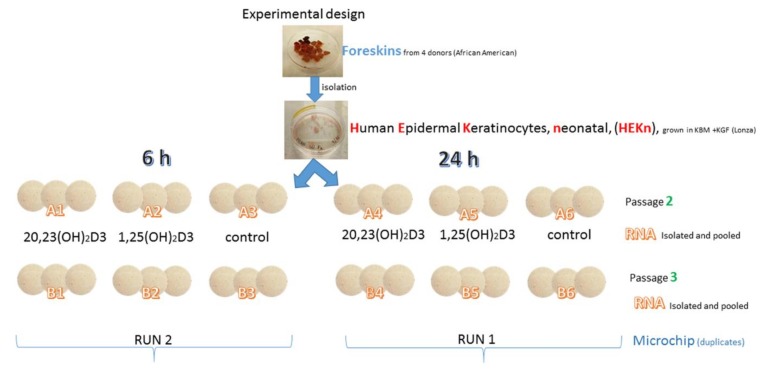
Outline of the experimental design.

The schematic outline of the experimental design is presented in Figure 2. Briefly, to test the divergence and overlap between the gene expression patterns, human neonatal epidermal keratinocytes combined from four African-American [79] donors were treated with 1,25(OH)_2_D3 or 20,23(OH)_2_D3 for 6 or 24 h. Microarray assays were performed using Illumina’s HumanWG-6_V2 (Platform GPL13376) chip/array as described in Materials and Methods and the raw data has been deposited at the NCBI GEO (GSE117351).

The relative changes in gene expression (average of two independent experiments that used triplicate cell cultures), were normalized vs. vehicle control (0.1% ethanol). Table 1 shows marked differences in the number of genes up- or downregulated by either 1,25(OH)_2_D3 or 20,23(OH)_2_D3 using 1.5-, 2- and 4-fold cut-off values (FC). Average signal values for filtered gene clusters with FC ≥ ±1.5 are shown in Supplemental excel file #1. Briefly, treatment with 1,25(OH)_2_D3 for 6 h leads to changes in the expression of 148 vs. 37 genes for 20,23(OH)_2_D3 when using 1.5-FC, and 38 vs. 21 and 3 vs. 0 when using 2- and 4-FC, respectively. After 24 h, this trend changed to 410 and 4079 genes regulated, respectively, by 1,25(OH)_2_D3 and 20,23(OH)_2_D3 with 1.5-FC value, and 119 and 1611 for 2-FC value and 12 and 199 genes for 4-FC value, respectively (Table 1).

**Figure 3:**
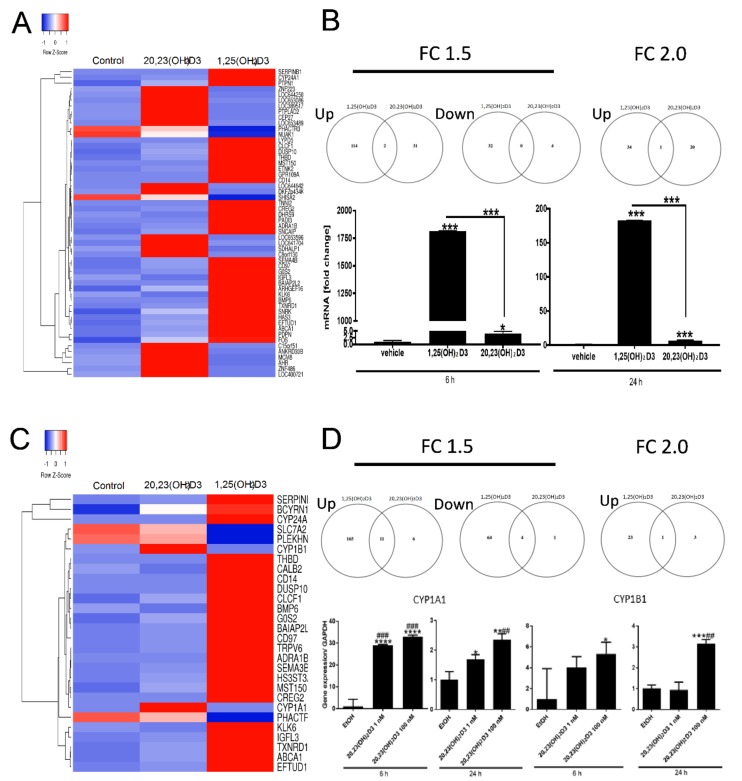
Changes in gene expression in human epidermal keratinocytes treated with 1,25(OH)_2_D3 or 20,23(OH)_2_D3 for 6 h. (**A**) Heat map of the gene expression pattern for experiment #1 with corresponding Venn diagrams for FC ≥ 2 and 1.5. (**B**) Effect of 10^−7^ M of 1,25(OH)_2_D3 or 20,23(OH)_2_D3 on *CYP24A1* expression in keratinocytes after 6 and 24 h treatment. (**C**) Heat map of the gene expression pattern for experiment #2 with corresponding Venn diagrams for FC-2 and 1.5. (**D**) Effect of 10^−9^ and 10^−7^ M of 20,23(OH)_2_D3 on CYP1A1 and CYP1B1 expression in keratinocytes after 6 or 24 h treatment. Data represent means ± SD (*n* = 3) where * *p* < 0.05, ** *p* < 0.01, *** *p* < 0.001 and **** *p* < 0.0001 at student *t*-test; ^##^ *p* < 0.01 and ^###^ *p* < 0.001 at one-way ANOVA test.

Hierarchical clustering identified patterns of genes responding to either 1,25(OH)_2_D3 or 20,23(OH)_2_D3, or to both. Selected gene clusters, representing the altered expression after 6 h of incubation as well as Venn diagrams are shown in Figure 3A. The heat maps corresponding to relative gene expression levels displayed both distinct or opposite, or common (overlapping) gene expression. For 2-FC there was only 1 common gene (CYP24A1) stimulated by both 1,25(OH)_2_D3 (82 fold) and 20,23(OH)_2_D3 (3.4 fold). This differential stimulation of CYP24A1 was further confirmed by qPCR (Figure 3B) and is consistent with the literature on 1,25(OH)_2_D3 [1,3,6,27,51] and 20,23(OH)_2_D3 [69,80]. For 1.5-FC there were two common genes, CYP24A1 and the gene with a target id ILMN_131812 (identified as small ILF3/NF90-associated RNA A1 (SNAR-A1)), for which expression was stimulated. 

Ingenuity pathway analysis using FC ≥ ±1.5 was performed. The top canonical pathway induced by 1,25(OH)_2_D3 was VDR/RXR signaling (Table 2) (Supplemental Figure S1A), which was expected [3,6,51,81,82]. This was followed by the roles of osteoblasts, osteoclasts and chondrocytes in rheumatoid arthritis; the role of macrophages, fibroblasts and endothelial cells in rheumatoid arthritis; and Toll-like receptor signaling (Table 2), which is consistent with previously reported functions of 1,25(OH)_2_D3 [3,6,30,31,45,51,54]. Interestingly, the next top nuclear receptor signaling pathway activated by 1,25(OH)_2_D3 was linked to the glucocorticoid receptor (GR) followed by the aryl hydrocarbon receptor [74], PPAR, PPARα/RXRα, LXR/RXR, and RAR (Table 2). The inclusion of these additional pathways could be secondary to the use of the same dimeric partner, RXR, and communication between receptors, or alternatively by activation by signaling pathways downstream of VDR. For example, it is already known that 1,25(OH)_2_D3 can selectively activate local elements of hypothalamo-pituitary adrenal axis in keratinocytes [71]. The significance of additional nuclear receptor signaling is out of the scope of this paper and is a goal of our future research. 

The top canonical nuclear receptor pathway induced by 20,23(OH)_2_D3 was AhR signaling (Supplemental Figure S2A) with VDR/RXR being next (Supplemental Figure S3A) (Table 3). While the identification of the VDR/RXR as the target for 20,23(OH)_2_D3 is consistent with previously reported functional data and molecular modeling [60,65,69,83], identification of the AhR as its primary target was unexpected and hence it was further analyzed in detail as described below. Table 4 shows that for 1,25(OH)_2_D3 the nuclear signaling pathways VDR/RXR, followed by AhR, PPARα/RXRα, RAR and LXR/RXR, were among the top toxicity-related pathways identified. The top signaling pathways for 20,23(OH)_2_D3 were linked to the activation of AhR and VDR/RXR (Table 5). Table 6 and Table 7 show certain functional similarities between top diseases and bifunctions affected by both molecules. For example, cancer, and organismal injury and abnormalities, are the top two diseases affected by both molecules. These phenotypic similarities are consistent with previously reported studies comparing the biological effects of 1,25(OH)_2_D3 and CYP11A1-derived D3-hydroxyderivatives, including 20,23(OH)_2_D3, and indicate similarities between the effects on cell proliferation and differentiation, as well as similar anti-inflammatory, photoprotective and anti-cancer actions [23,60,61,62,64,72,80,84].

Because of the unexpected differences between 1,25(OH)_2_D3 and 20,23(OH)_2_D3, the 6 h incubation experiment was repeated in a similar manner as shown in Figure 2 and microarray analyses were performed using Illumina’s HumanWG-6_V2 (Platform GPL13376) chip/array. Average signal values for filtered gene clusters with FC ≥ ±1.5 are shown in Supplemental excel file #2. The heat maps corresponding to relative gene expression and Venn diagrams are shown in Figure 3C. Again, for a 2-fold cut-off value there was only one common gene (*CYP24A1*) whose expression was stimulated by both 1,25(OH)_2_D3 (80-fold) and 20,23(OH)_2_D3 (2.9-fold). For FC ≥ ±1.5 there were 11 common genes upregulated and 4 downregulated. Again, ingenuity pathway analysis showed that VDR/RXR was the top canonical pathway induced by 1,25(OH)_2_D3, followed by the role of osteoblasts, osteoclasts and chondrocytes in rheumatoid arthritis. As before, other nuclear receptor signaling pathways included LXR/RXR, GR and AhR. For 20,23(OH)_2_D3 the top nuclear receptor signaling pathways were again AhR and VDR/RXR. Of note, this microarray showed that 20,23(OH)_2_D3 upregulated two genes downstream of AhR signaling, *CYP1A1* and *CYP1B1*, by factors of 2.4 and 2.6, respectively. This stimulation was confirmed by qPCR (Figure 3D). VDR/RXR was identified as the top toxicity pathway for 1,25(OH)_2_D3 and AhR for 20,23(OH)_2_D3.

**Figure 4:**
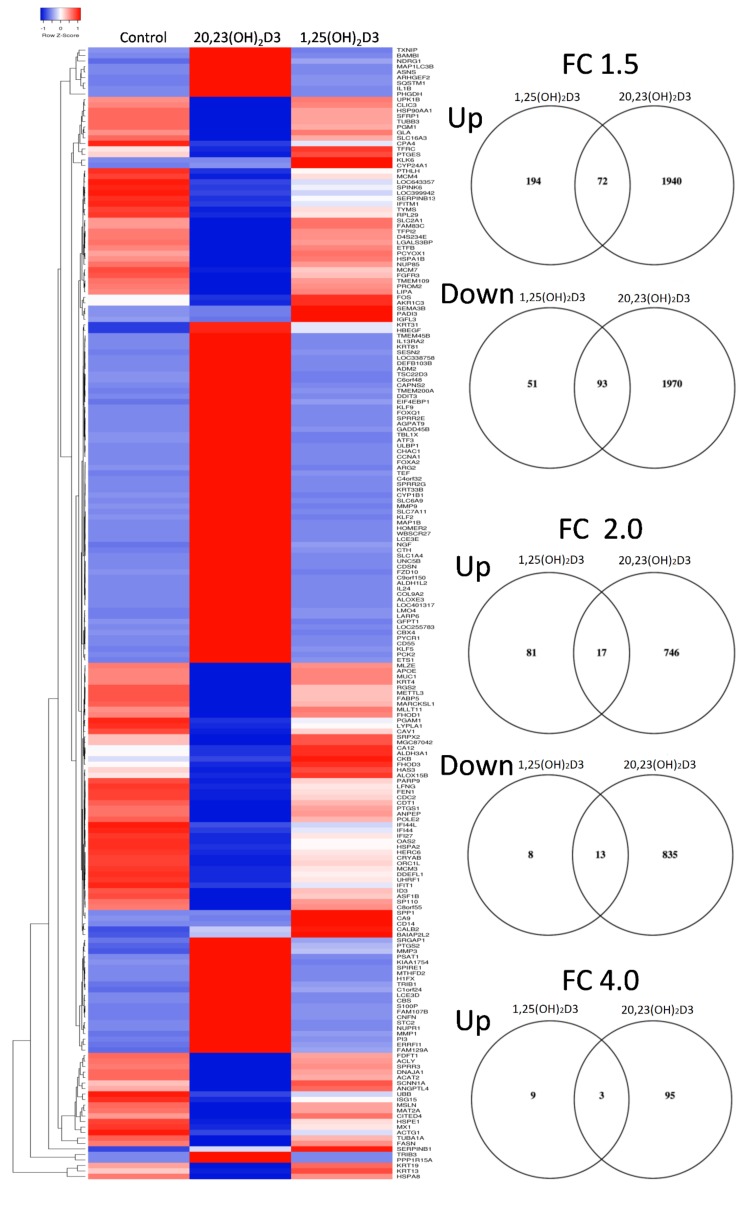
Heat map of gene expression pattern in human epidermal keratinocytes treated with 10^−7^ M of 1,25(OH)_2_D3 or 20,23(OH)_2_D3 for 24 h. On the right are the corresponding Venn diagrams for FC ≥ 4, 2 and 1.5.

More robust data were obtained with 24 h of treatment for which the average signal values for filtered gene clusters with FC ≥ ±1.5 are shown in Supplemental excel file #3. Because of the large number of genes affected (Table 1), the heat map of differentially expressed genes and Venn diagrams were generated using the 4-FC value which show three overlapping genes (*CYP24A1*, *MMP3* and *SERPINB1*) as well as distinct gene expression patterns (Figure 4). For FC ≥ ±1.5, 93 and 72 common genes were up- and downregulated, respectively. Ingenuity pathway analysis using FC ≥ ±2.0 was consistent with results obtained after 6 h of treatment. Again, the top canonical pathway for 1,25(OH)_2_D3 was VDR/RXR (Supplemental Figure S1B) followed by MIF-related glucocorticoid regulation and regulation of the innate immunity system (Table 8). AhR signaling was also listed. The top canonical pathways induced by 20,23(OH)_2_D3 were AhR signaling (Supplemental Figure S2B) and the cholesterol biosynthesis pathway (Table 9). Interestingly, the involvement of a second nuclear receptor complex was emphasized by VDR/RXR activation (Supplemental Figure S3B), with p53 signaling also being listed. The latter is consistent with the photoprotective properties of 20,23(OH)_2_D3 and activation of p53 by its direct precursor, 20(OH)D3 [23]. The top affected toxicity pathways for 1,25(OH)_2_D3 included VDR/RXR, xenobiotic metabolism, cardiac fibrosis and cytochrome P450s (Table 10). AhR signaling was also listed. For 20,23(OH)_2_D3, AhR signaling was again listed as the top toxicity pathway followed by cholesterol synthesis, p53 signaling and again VDR/RXR (Table 11). The top upstream gene regulation pathways for 1,25(OH)_2_D3 included vitamin D3-VDR-RXR, calcitriol, dexamethasone, progesterone and β-estradiol, while for 20,23(OH)_2_D3, included TP53 (p53 tumor suppressor), β-estradiol, lipopolysaccharide, TNF (tumor necrosis factor) and TGF β1 (transforming growth factor-β1) (not shown).

Table 12 and Table 13 show some similarities and differences with dermatological diseases and conditions; with cancer, organismal injury and abnormalities being the main diseases affected by 20,23(OH)_2_D3 and 1,25(OH)_2_D3. With regard to molecular and cellular functions, cellular growth and proliferation, cell death and survival, cellular movement and cell cycle were the major functions for 20,23(OH)_2_D3, and cellular movement, cell signaling, small molecule biochemistry, lipid metabolism and cellular development for 1,25(OH)_2_D3. Among the 25 networks activated by 20,23(OH)_2_D3, the top five included: (1) connective tissue disorders, neurological diseases, organismal injuries and abnormalities, (2) RNA post-transcriptional modification, carbohydrate metabolism and lipid metabolism, (3) connective tissue, developmental, skeletal and muscular disorders, (4) cellular movement, endocrine system disorders, gastrointestinal diseases and (5) nucleic acid metabolism, small molecules biochemistry and dermatological diseases and conditions. Among the 15 networks activated by 1,25(OH)_2_D3, the top five included: (1) cancer, organismal functions, organismal injuries and abnormalities, (2) cell-to-cell signaling and interaction, cellular assembly and organization, cellular development, (3) cellular growth and proliferation, tissue development and cancer, (4) molecular transport, carbohydrate and lipid metabolism and (5) protein degradation, protein synthesis, cellular assembly and organization. 

**Figure 5:**
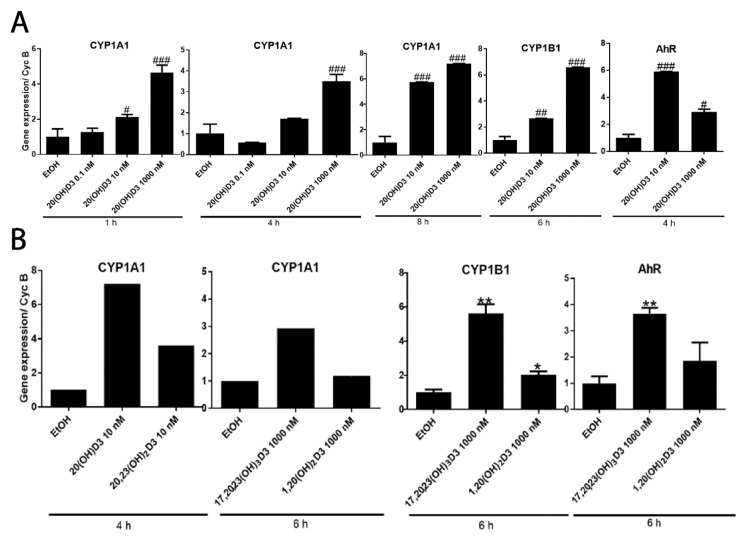
Changes in CYP1A1, CYP1B1 and AhR gene expression in HaCaT keratinocytes treated with vitamin D3 hydroxyderivatives as a function of the time of treatment. **A.** Dose-dependent effect of 20(OH)D3 on the gene expression. Data represent means ± SD (*n* = 3) where ^#^ *p* < 0.05, ^##^ *p* < 0.01 and ^###^ *p* < 0.001 at one-way ANOVA test. **B.** Effect of 1,25(OH)_2_D3 and 20,23(OH)_2_D3 on the gene expression as indicated. Data represent means (*n* = 2) for CYP1A1, or means ± SD (*n* = 3) for CYP1B1 and AhR where * *p* < 0.05, ** *p* < 0.01, at student *t*-test.

**Figure 6:**
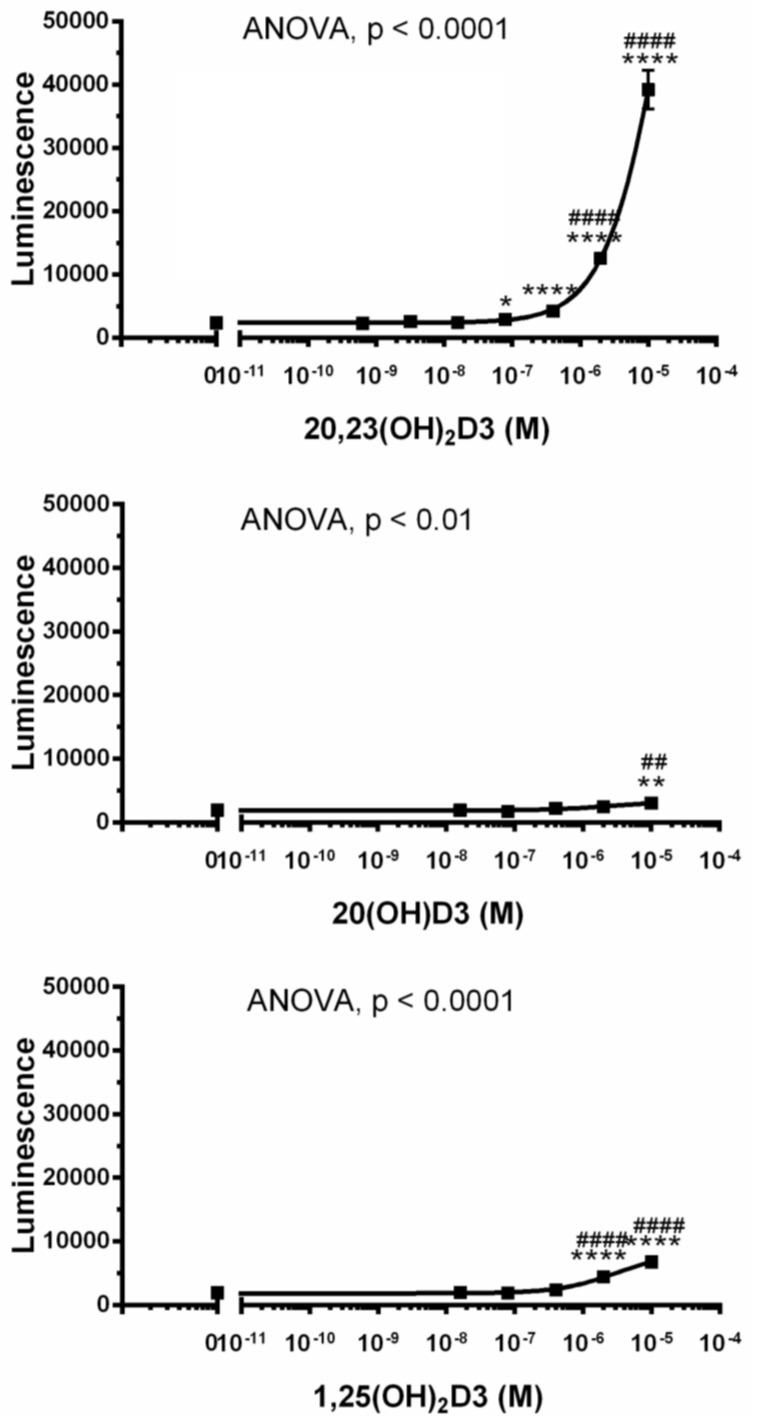
Stimulation of AhR activity by 20(OH)D3, 22,23(OH)_2_D3, and 1,25(OH)_2_D3. The assays for 22,23(OH)_2_D3 were performed in quadruplicate, while for 20(OH)D3 and 1,25(OH)_2_D3 in triplicate. Data represent means ± SD where * *p* < 0.05, ** *p* < 0.01 and **** *p* < 0.0001 at student *t*-test; ^##^ *p* < 0.01 and ^####^ *p* < 0.0001 at one-way ANOVA test.

Because of the unexpected finding that AhR signaling represented the top regulatory pathway activated by 20,23(OH)_2_D3, and is validated by qPCR analysis of *CYP1A1* and *CYP1B1* genes expression (Figure 3D), we examined whether 20(OH)D3, which is the precursor to 20,23(OH)_2_D3, and 17,20,23(OH)_2_D3 and 1,20(OH)_2_D3, which are downstream metabolites (see Figure 1), also affected the expression of genes linked to AhR in HaCaT keratinocytes. Figure 5A shows that 20(OH)D3 stimulated the expression of *CYP1A1* and *CYP1B1* in a dose-dependent fashion, with a stimulatory effect also seen for the *AhR* gene. 17,20,23(OH)_3_D3 (1 µM) could also stimulate *CYPA1*, *CYP1B1* and *AhR* expression, while 1,20(OH)_2_D3 had only a small effect on *CYPB1* and no effect on *CYP1A1* and *AhR*. Finally, we used a Human AhR Reporter Assay System (INDIGO, Biosciences) to analyze the effect of several D3-hydroxyderivatives on AhR-mediated transactivation. The kit contains AhR Reporter Cells that contain the luciferase reporter gene functionally linked to an AhR-responsive promoter, which provides a sensitive surrogate measure of the changes in AhR-mediated activation of luciferase reporter. Figure 6 shows that there was marked activation of AhR activity by 20,23(OH)_2_D3 with weaker but significant activation by 20(OH)D3 or 1,25(OH)_2_D3. Thus, the functional studies support the microarray analysis indicating that hydroxyderivatives of D3 can act on AhR. This finding can be explained by the promiscuous nature of AhR and its activity [85].

**Figure 7:**
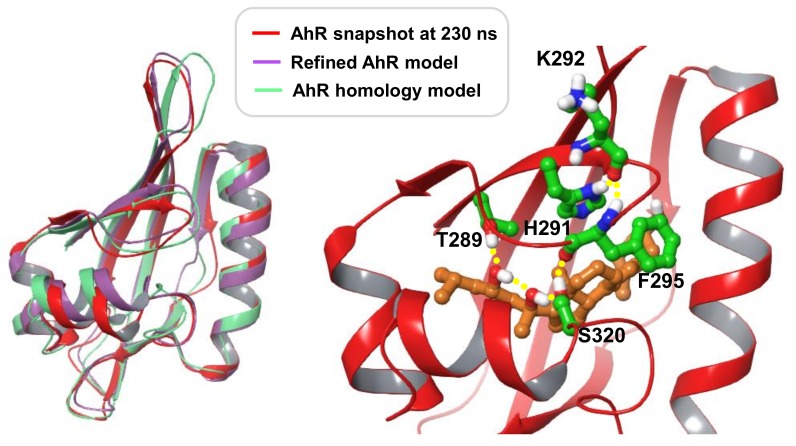
Structural fold of AhR models. To the left: Superimposed are the initial homology model, the refined AhR model and a molecular dynamic simulation snapshot at 230 ns. To the right: Close-up view of the simulation snapshot at 230 ns, displaying the ligand and AhR residues involved in an interaction network, as discussed in the text. 20*S*,23*S*(OH)_2_D3 is shown with carbon atoms colored light brown, AhR residue carbons colored green; all other atoms are colored by atom type (O: red, N: blue, S: yellow). Hydrogen bonding interactions are indicated with yellow spheres.

An additional mechanistic insight into the above interactions was provided by modeling using the crystal structure of the ligand-binding domain (LBD) of human AhR. The presently available crystal structure of the human AhR (PDB: 5NJ8) is missing the LBD region. A model of the human AhR LBD with bound 20S,23S(OH)_2_D3 was developed as described under Methods. Briefly, the final model was based on the homology modelling template of C-terminal Per-ARNT-Sim domain of Hypoxia-Inducible Factor-2α, PDB entry code 3H82. The sequence identity between human AhR and the modelled sequence is 27%; the alignment is shown in Supplemental Figure S4. Short molecular dynamic simulation runs were performed on selected docked poses of 20S,23R/S(OH)_2_D3 epimers in order to identify binding modes most favorably accommodated in the binding site of the homology model. The selected complex with 20S,23S(OH)_2_D3 was simulated for 100 ns to allow for local structural adjustments of flexible regions to the presence of the vitamin D3 scaffold. The final conformer obtained is referred to as the ‘refined AhR model’. Further ligand-induced effects were explored through a 250 ns simulation production run starting with this model. Over the first 130 ns the ligand-induced conformational changes were in the vicinity of F295 and S320. The latter is in a flexible region with two adjacent glycine residues while F295 is part of a loop structure ‘covering’ the binding pocket. The conformation adopted by 130 ns in these regions were maintained for the rest of the simulation time, likely stabilized by a hydrogen bonding network that formed, involving ligand hydroxyl groups, T289, S320 side chains and the backbone of F295 as shown in a representative simulation snapshot at 230 ns in Figure 7. Interactions of this network link the more rigid beta-sheet structure of the pocket containing T289 with two loop regions. The flexible ‘belt’ between G309-H326 includes a short helical segment near S320 that also shifted due to the presence of the ligand. This binding mode also changes the preferred orientation of H291 which by 130 ns simulation time forms a stable hydrogen bond with the backbone carbonyl of K292, an interaction not present in the initial or refined AhR models. Alanine mutation of T281, H285 of mouse AhR corresponding to the human residues T289 and H291 was shown to dramatically decrease Hsp90 binding [86]. Figure 7 illustrates that differences in the structural fold of AhR between the homology model, the refined AhR model and the simulation conformer are mainly within loops and the flexible ‘belt’ region.

**Figure 8:**
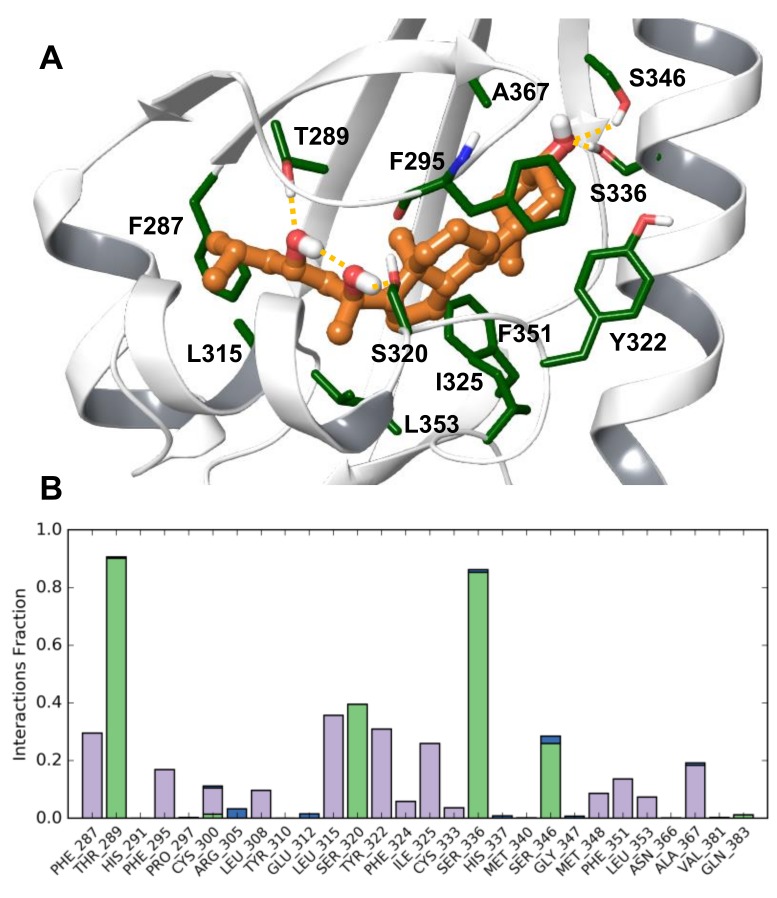
(**A**) The proposed binding model 20*S*,23*S*(OH)_2_D3 at human AhR. (**A**) Representative simulation snapshot at 230 ns. Shown residues contribute to the binding of the ligand over simulation time. (**B**) Fraction of simulation time during which interactions are present with each AhR residue, averaged over 130–250 ns of the simulation production run.

The proposed binding model of 20S,23S(OH)_2_D3 is shown in Figure 8A through a representative simulation conformer at 230 ns. Figure 8B shows the fraction of simulation time during which interactions are present with each AhR residue, as averaged over 130—250 ns. The most stable polar interactions are hydrogen bonding between 23-OH and T289 at 90% and between 3-OH and S336 at 85%, with S346 also contributing 26% of the simulation time. These interactions anchor the two end regions of the scaffold in the pocket. 20-OH is hydrogen bonding with S320 for 39% of the stimulation time. Due to intra-molecular hydrogen bonding between the ligand hydroxyls, the 20-OH group is positioned to act as a hydrogen bond donor to S320, which allows S320 to interact with F295. This interaction is likely important for these loop conformational changes and their effect on H291. Ligand–protein contacts versus simulation time are shown in Supplemental Figure S5.

**Figure 9:**
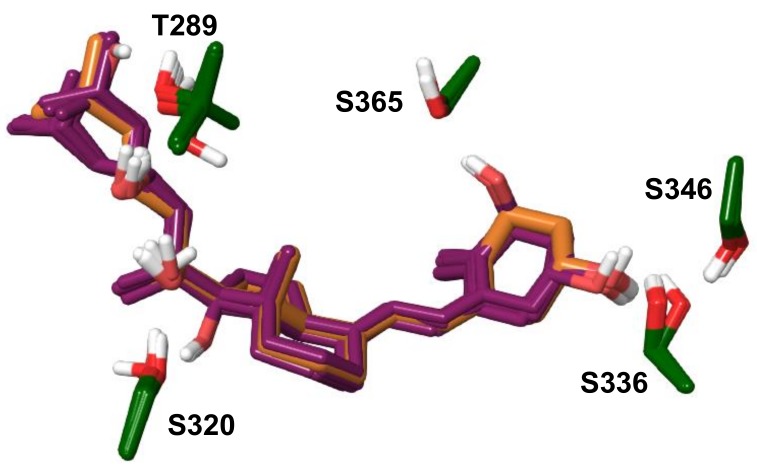
Induced Fit docked vitamin D3 analogs displayed simultaneously. The pose of 20*S*,23*S*(OH)_2_D3 from the refined AhR model is shown for comparison, with carbon atoms colored light brown. Only AhR residues involved in polar interactions are shown.

Loop conformational changes induced by 20S,23S(OH)_2_D3 are likely specific to this ligand. Therefore, for docking other vitamin D3 analogs the refined AhR model was utilized, applying the Induced Fit method. As shown in Table 14, Glide XP docking scores of three analogs are notably lower compared to other compounds within this set, 20(OH)D3, 1,25(OH)_2_D3 and 1,20(OH)_2_D3. Docked poses of all analogues are very similar, and also closely overlap with 20S,23S(OH)_2_D3 in the refined AhR model. Docked poses for all analogs are displayed in Figure 9, along with the binding mode of 20S,23S(OH)_2_D3 for comparison. Residues from Induced Fit structures contributing to polar interactions with ligands are shown only; all residues in proximity of docked ligands are included in Supplemental Figure S6. Docked Vitamin D3 analogs share similar hydrogen bonding interactions through hydroxyl groups: 1-OH interacts with S365, 3-OH with S336 and possibly S346, 17-OH and 20-OH with S320, 23-OH with T289. Docking results predict that 25-OH interacts with T289. A short, 20 ns molecular dynamic simulation was performed on 20(OH)D3, 1,25(OH)_2_D3, 17,20,23S(OH)_3_D3, starting with docked poses. The ligands maintained the binding mode and predicted interactions during simulation except for 1,25(OH)_2_D3. Therefore, simulation of the latter was extended another 50 ns, during which the pose of 1,25(OH)_2_D3 shifted, disrupting hydrogen bonding between 3-OH and S336 that was only present for 35% of simulation time. In comparison, in the case of 20(OH)D3 and 17,20,23S(OH)_3_D3 the same interaction was present 85% and 66% of time, respectively. Due to mobility of the aliphatic chain in the binding site, 25-OH formed contacts with T289 30% and Y310 37% of the simulation time. 1,25(OH)_2_D3 may have a distinct binding mode and interaction with AhR than the other analogs. Interactions of 17,20,23S(OH)_3_D3 are analogous to those of 20*S*,23*S*(OH)_2_D3. However, hydrogen bonding between 17-OH with S320 may interfere with structural changes such as those induced by 20*S*,23*S*(OH)_2_D3 during the 250 ns simulation production run. While 20(OH)D3 is also predicted to form analogous contacts, the absence of 23(OH) interactions is likely significant.

### Modelling Conclusions

Molecular dynamic simulation of the developed AhR-20*S*,23*S*(OH)_2_D3 model predicts strong hydrogen bonding interactions between this ligand and T289, S336. A hydrogen bond formed with S320 is also well maintained during simulation. A number of AhR residues have favorable non-polar contacts with the ligand (Figure 8). The simulation trajectory predicts that ligand-specific interactions induce a conformational change in the region in the vicinity of S320 and F295, also leading to a distinct position and interaction of H291. The interaction network that forms during simulation due to the ligand links the beta-sheet structure of the pocket with two loops, restraining the conformation of flexible regions in the binding site. The presented model is also consistent with the observed effect of 20*S*,23*S*(OH)_2_D3 on AhR since, in particular, T289 and H291 are essential residues for Hsp90 binding. 

Docking of a set of D3 analogs predicts ligand binding modes close to that of 20*S*,23*S*(OH)_2_D3, as well as analogous interactions with AhR. Short simulation runs of docked poses of 20(OH)D3 and 17,20,23*S*(OH)_3_D3 predict stability of the starting ligand poses. While forming interactions analogous to those of 20*S*,23*S*(OH)_2_D3, these two analogs lack features that contribute to the induced effects of 20*S*,23*S*(OH)_2_D3 during simulation. The 20(OH)D3 analog lacks hydrogen bonding through 23-OH and in the case of 17,20,23*S*(OH)_3_D3 the 17-OH group may interfere with the interactions between S320 and the F295 backbone. Stability of the docked pose of 1,25(OH)_2_D3 was also explored through molecular dynamic simulation. Shifting and fluctuations of 1,25(OH)_2_D3 over simulation time suggests that this ligand would not adopt a binding mode close to that of 20*S*,23*S*(OH)_2_D3 in the AhR binding site. Thus, modelling predictions are consistent with the distinct effects of these D3 analogs on AhR.

## 3. Materials and Methods

### 3.1. Materials

Vitamin D3 (D3) and 1,25(OH)_2_D3 were purchased from Sigma-Aldrich (St. Louis, MO, USA). 20,23(OH)_2_D3 was produced by hydroxylation of D3 by CYP11A, extracted with dichloromethane and purified as described in References [14,66]. 20*S*-Hydroxyvitamin D3 (20(OH)D3, 1α,20*S*-dihydroxyvitamin D3 (1,20(OH)_2_D3) and 17,20S,23*S*-trihydroxyvitamin D3 (17,20,23(OH)_3_D3) were also synthesized using CYP11A1 as described before [66,87]. An extinction coefficient of 18,000 M^−1^ cm^−1^ at 263 nm was used to quantify concentrations of 20,23(OH)_2_D3 [88] and the secosteroids were divided, dried and stored at −80 °C until use. Secosteroids were dissolved in ethanol prior to experiments to obtain stock solutions of 10^−4^ M.

The structures of the secosteroids tested and the routes of enzymatic synthesis that include C25 and C1 hydroxylation for 1,25(H)_2_D3 [5], and the sequential hydroxylation of the D3 side chain by CYP11A1 producing 20,23(OH)_2_D3 and 17,20.23(OH)_3_D3 [14,66], are shown in Figure 1. 

### 3.2. Cell Culture

Neonatal foreskins of African American [79] donors were used to isolate neonatal human epidermal keratinocytes (HEK) following standard protocols described previously [69,89]. The use of human tissues were approved both by the IRB at the UTHSC as an exempt protocol #4 and by the IRB at the University of Alabama Birmingham, as they are not subject to FDA regulation and not Human Subject Research. Cells were grown in keratinocyte basal medium (KBM) supplemented with keratinocyte growth factors (KGF) (Lonza, Walkersville, MD, USA) on collagen coated plates [68] and second and third passages were used for the experiments [69]. Human epidermal HaCaT keratinocytes were cultured in Dulbecco’s Modified Eagle Medium (DMEM) supplemented with glucose, l-glutamine, pyridoxine hydrochloride (Cell Grow), 5% fetal bovine serum (FBS) (Atlanta Biologicals, Flowery Branch, GA, USA) and 1% penicillin/streptomycin/amphotericin antibiotic solution (Thermo Fisher Scientific, Waltham, MA, USA). Human cells were cultured at 37 °C, with a CO_2_ concentration of 5%, 100% humidity, and media were changed every second and/or third day.

Prior to treatment with secosteroids, HaCaT cells were serum deprived for 24 h and the medium was changed to DMEM medium containing 5% charcoal-treated FBS (ctFBS) (Atlanta Biologicals, Flowery Branch, GA, USA) to which D3 hydroxymetabolites from the stock solutions were added. For epidermal neonatal keratinocytes, the KBM with KGF was supplemented with 0.5% bovine serum albumin (BSA) prior to the addition of D3 derivatives.

### 3.3. Microarray Assays

Petri dishes (100 mm in diameter) were seeded with human neonatal keratinocytes that were combined from five different black donors at either passage 2 or 3. After reaching 70–80% of confluence, cells were treated with 10^−7^ M of either 20,23(OH)_2_D3 or 1,25(OH)_2_D3, or with 0.1% ethanol (EtOH) as a solvent control for 6 or 24 h. After, these cells were isolated from three plates per each experimental condition and combined for passage 2 and 3, separately (Figure 2).

The RNA from HEK treated with either 20,23(OH)_2_D3 or 1,25(OH)_2_D3, or 0.1% ethanol control, was isolated using the Absolutely RNA Miniprep Kit (Qiagen, Germantown, MD, USA). High purity RNA samples were subjected to microarray analysis at the Molecular Resources Center at the UTHSC. Expression profiling was accomplished using whole-genome gene expression direct hybridization assay using Illumina’s HumanWG-6_V2 (Platform GPL13376) chip/array (Illumina, San Diego, CA, USA). Each array contains full-length 50-mer probes representing more than 22,000 well-annotated RefSeq transcripts, including up-to-date genes derived from the National Center for Biotechnology Information Reference Sequence (NCBI RefSeq) database. Initially, 250 ng total RNA was converted to cDNA, followed by an in vitro transcription step to generate labeled cRNA following the manufacturer’s recommendations (Applied Biosystems, Foster City, CA, USA).

The labeled probes were then mixed with hybridization reagents and hybridized overnight to the Human BeadChips. Following washing and staining, the BeadChips were imaged using the Illumina BeadArray Reader to measure fluorescence intensity at each probe. The intensity of the signal corresponds to the quantity of the respective mRNA in the original sample.

### 3.4. Bioinformatics Analysis

For generating networks, a data set containing gene identifiers and corresponding expression values was uploaded into the application. Each identifier was mapped to its corresponding object in Ingenuity’s Knowledge Base. A FC of ±2 or ±1.5, where indicated, was set to identify molecules whose expression was significantly differentially regulated. These molecules, called Network Eligible molecules, were overlaid onto a global molecular network developed from information contained in Ingenuity’s Knowledge Base. Networks of Network Eligible Molecules were then algorithmically generated based on their connectivity. The Functional Analysis identified the biological functions and/or diseases that were most significant to the entire data set. Molecules from the dataset that met the FC cutoff of ±2 or ±1.5 and were associated with biological functions and/or diseases in Ingenuity’s Knowledge Base were considered for the analysis. Right-tailed Fisher’s exact test was used to calculate a p-value determining the probability that each biological function and/or disease assigned to that data set is due to chance alone.

### 3.5. Real-Time Reverse Transcription Polymerase Chain Reaction (qRT-PCR)

Semiconfluent cultures of human neonatal keratinocytes or HaCaT cells were treated for 6 h, 24 h or as indicated in the figure legends with vitamin D3 hydroxyderivatives or ethanol, and RNA isolated as described above. Reverse transcription was done using the Transcriptor First Strand cDNA Synthesis Kit (Applied Biosystems, Foster City, CA, USA) with 100 ng RNA per reaction. qRT-PCR was performed using cDNA diluted 10-fold in sterile water and a TaqMan PCR Master Mix. Reactions (in triplicate) were performed at 50 °C for 2 min, 95 °C for 10 min and then 50 cycles of 95 °C for 15 s and 60 °C for 1 min. The primers and probes were designed with the universal probe library (Roche). Data were collected on a Roche Light Cycler 480. The amount of amplified product for each gene was compared to that of Cyclophilin B or GAPDH using a comparative C_T_ (ΔΔC_T_) method. Supplemental Table S1 lists the primers used for qRT-PCR amplifications.

### 3.6. Interaction of Hydroxyvitamin D Derivatives with AhR

Interaction of 20(OH)D3, 20,23(OH)_2_D3 and 1,25(OH)_2_D3 with AhR was evaluated using the Human AhR Reporter Assay System (INDIGO Biosciences, State College, PA, USA) according to the manufacture’s protocol. Briefly, AhR reporter cells were recovered on a 96-well plate frame using the cell recovery medium for 5 h, followed by treatment with vitamin D3 hydroxyderivatives in the compound screening medium for 22 h. After removing the media from the wells, luciferase detection reagent was added to the wells and luminescence was measured using a Cytation 5 Cell Imaging Multi-Mode Reader (Winooski, VT, USA).

### 3.7. Statistical Analyses

Data are presented as means ± SD (*n* = 3–4), and were analyzed with a Student’s *t*-test (for two groups) or ANOVA using Prism 4.00 (GraphPad Software, San Diego, CA, USA). Statistically significant differences are denoted with asterisks for *t*-tests or for one way ANOVA with # as indicated in the figure legends.

### 3.8. Data Deposition

The data reported in this paper have been deposited in the Gene Expression Omnibus (GEO) database, https://www.ncbi.nlm.nih.gov/geo (accession no. GEO: GSE117351).

### 3.9. Development of a Human AhR LBD Model Complexed with 20S,23S(OH)_2_D3

The strategy applied utilized tools implemented in the Schrödinger software package, version 2017-4 (Schrödinger, LLC, New York, NY, USA). Homology modelling of the human AhR ligand-binding domain was based on crystal structures of the C-terminal Per-ARNT-Sim domain of Hypoxia-Inducible Factor-2α (HIF-2α PAS-B), PDB entry codes 3H82 and 4XT2. Based on the two templates, two homology models were built using the energy-based homology model building method in Schrödinger. The sequence identity is 27% between human AhR and template sequences in the modelled LBD region. The sequence alignment is shown in Supplemental Figure S4. Residues were numbered according to the human AhR sequence (Uniprot ID P35869). Co-crystallized ligands were included. Modeled loops that contained gaps in the sequence alignment were refined through default loop refinement options. The models were relaxed through restrained energy minimization in Protein Preparation Wizard (OPLS3 force field).

Initial binding mode hypotheses were generated through docking 20*S*,23*R*/*S*(OH)_2_D3 into the two obtained AhR models. Out of the top scoring poses at both AhR models, eight were selected for protein-ligand complex refinement (Prime tool in Schrödinger software), followed by a 10 ns molecular dynamic simulation run for each complex using Desmond. Four poses induced distortions in rigid, AhR beta-sheet/helical backbone structures within 10 ns simulation and were not considered further. Out of the remaining poses, the most favorable contacts were formed by two similar poses of the epimers: 20*S*,23*S*/*R*(OH)_2_D3. Simulation of these poses was extended to 20 ns, which suggested that 20*S*,23*S*(OH)_2_D3 is more favorably accommodated than its *R* epimer. The 20*S*,23*S*(OH)_2_D3 complex conformer at 20 ns showed an overall RMSD (root-mean-square deviation) of 1.48 from its homology modelling template (PDB: 3H82). In order to allow flexible regions to adjust to the presence of the bound vitamin D3 scaffold, the model was further simulated for 100 ns or for 230 or 25 ns as indicated. The final conformer was relaxed through restrained energy minimization and is referred to as the ‘refined AhR model’. The overall RMSD of this model from its homology modelling template (PDB: 3H82) is 1.58, suggesting stability of the AhR structure over simulation time. The model structure contains only two residues with backbone dihedrals in disallowed regions, both of which are glycines: Gly309 and Gly374.

### 3.10. Docking Method

The induced Fit docking method was used as implemented in Schrödinger, version 2017-4 (Schrödinger, LLC, New York, NY, USA). This method combines Prime tools and Glide docking, taking into account the flexibility of residues in proximity to the ligand. 20,23(OH)_2_D3 was Induced Fit docked into the two human AhR homology models using default parameters except optimization of side chains was extended to 6 Å around the ligand and Glide re-docking was done in extra-precision mode. Docking of vitamin D3 analogs into the refined AhR model also utilized Induced Fit with default options, except that van der Waals scaling parameters for docking were set to 1.0 for both protein and ligand (no scaling), and re-docking was done in extra-precision mode.

### 3.11. Molecular Dynamic Simulation Method

Molecular dynamic simulations were performed using Desmond (Schrödinger, LLC, New York, NY, USA) with the OPLS3 force field. Structures were solved in TIP3P explicit waters with boundary conditions in a 10 Å buffered orthorhombic system. Counter-ions were added. The NPT ensemble was employed with temperature fixed at 300 K and pressure at 1.01 bar. The cutoff radius for Coulombic interactions was set to 10 Å. The trajectory was recorded at 10 ps intervals.

## 4. Conclusions

Gene expression profile analysis demonstrated that 20,23(OH)_2_D3 and 1,25(OH)_2_D3 induce distinct and overlapping gene expression patterns in keratinocytes linked to the activation of common (VDR-dependent) and distinct (involving other nuclear receptors) signal transduction pathways. Taking into consideration the strong chemical similarity between 20,23(OH)_2_D3 and 1,25(OH)_2_D3 (Figure 1), the marked differences in gene expression panels (Table 1, Figure 3 and Figure 4) were unexpected. This is because our previous studies predominantly showed phenotypic similarities between the effects of both dihydroxy-vitamin D3 species, such as regulation of cell proliferation and differentiation, and anti-inflammatory, photoprotective and anticancer functions, with only a couple of notable differences where 20,23(OH)_2_D3 displayed no calcemic effects and poor activation of CYP24A1. These predominantly overlapping effects are most likely secondary to the redundancy of downstream phenotypic regulators and intercommunication between distinct transduction pathways at the cell or organ levels. The similarities are likely related to the activation of the VDR. The most significant and unexpected finding is the identification of AhR as the major receptor for 20,23(OH)_2_D3, which also appears to be activated by other CYP11A1-derived vitamin D3 derivatives, and possibly by 1,25(OH)_2_D3 to some extent, as predicted by molecular modeling. The future challenge is to precisely define the interaction of different vitamin D3 hydroxyderivatives with the ligand binding domain of AhR and how it is affected by the location of the OH-group on the side chain or at C1α, and how the activation of downstream signal transduction pathways occurs.

## Figures and Tables

**Table 1:** Number of genes up or downregulated in keratinocytes by 1,25(OH)_2_D3 or 20,23(OH)_2_D3 in comparison to vehicle control using 1.5-, 2- and 4-fold cut-off values.

Time	Genes	1,25(OH)_2_D3			20,23(OH)_2_D3		
		>1.5-Fold	>2-Fold	>4-Fold	>1.5-Fold	>2-Fold	>4-Fold
6 h	Upregulated	116	35	3	33	21	0
	Downregulated	32	3	0	4	0	0
24 h	Upregulated	266	98	12	2013	763	98
	Downregulated	144	21	0	2066	848	101

**Table 2:** Canonical pathways activated by 1,25(OH)_2_D3 in human epidermal keratinocytes after 6 h of treatment. Nuclear receptors are marked in bold.

Ingenuity Canonical Pathways	*p*-Value	Overlap (%)	Downregulated	No Change	Upregulated	No Overlap with Dataset
**VDR/RXR Activation**	1.0 ×10^−10^	12.8	34/78 (44%)	0/78 (0%)	43/78 (55%)	1/78 (1%)
Role of Osteoblasts, Osteoclasts and Chondrocytes in Rheumatoid Arthritis	2.2 × 10^−7^	5.02	100/219 (46%)	0/219 (0%)	114/219 (52%)	5/219 (2%)
Role of Macrophages, Fibroblasts and Endothelial Cells in Rheumatoid Arthritis	4.4 × 10^−6^	3.72	144/296 (49%)	0/296 (0%)	138/296 (47%)	14/296 (5%)
Toll-like Receptor Signaling	9.3 × 10^−6^	8.11	39/74 (53%)	0/74 (0%)	33/74 (45%)	2/74 (3%)
Hepatic Cholestasis	1.2 × 10^−5^	4.94	83/162 (51%)	0/162 (0%)	75/162 (46%)	4/162 (2%)
**Glucocorticoid Receptor Signaling**	1.5 × 10^−5^	3.64	152/275 (55%)	0/275 (0%)	117/275 (43%)	6/275 (2%)
Role of Cytokines in Mediating Communication between Immune Cells	3.1 × 10^−5^	9.09	32/55 (58%)	0/55 (0%)	20/55 (36%)	3/55 (5%)
IL-10 Signaling	8.5 × 10^−5^	7.35	33/68 (49%)	0/68 (0%)	34/68 (50%)	1/68 (1%)
IL-6 Signaling	0.00012	5.17	57/116 (49%)	0/116 (0%)	59/116 (51%)	0/116 (0%)
p38 MAPK Signaling	0.000126	5.13	62/117 (53%)	0/117 (0%)	52/117 (44%)	3/117 (3%)
MIF Regulation of Innate Immunity	0.000151	9.76	19/41 (46%)	0/41 (0%)	21/41 (51%)	1/41 (2%)
Molecular Mechanisms of Cancer	0.000155	2.74	201/365 (55%)	0/365 (0%)	157/365 (43%)	7/365 (2%)
iNOS Signaling	0.0002	9.09	23/44 (52%)	0/44 (0%)	20/44 (45%)	1/44 (2%)
**Aryl Hydrocarbon Receptor Signaling**	0.000331	4.29	73/140 (52%)	0/140 (0%)	61/140 (44%)	6/140 (4%)
**PPAR Signaling**	0.000398	5.32	52/94 (55%)	0/94 (0%)	38/94 (40%)	4/94 (4%)
LPS/IL-1 Mediated Inhibition of RXR Function	0.000631	3.2	119/219 (54%)	0/219 (0%)	88/219 (40%)	12/219 (5%)
TNFR2 Signaling	0.000891	10.3	14/29 (48%)	0/29 (0%)	14/29 (48%)	1/29 (3%)
HMGB1 Signaling	0.001202	4.17	54/120 (45%)	0/120 (0%)	63/120 (53%)	3/120 (3%)
MIF-mediated Glucocorticoid Regulation	0.001318	9.09	16/33 (48%)	0/33 (0%)	16/33 (48%)	1/33 (3%)
ILK Signaling	0.001479	3.23	102/186 (55%)	0/186 (0%)	77/186 (41%)	7/186 (4%)
IL-17A Signaling in Fibroblasts	0.001549	8.57	20/35 (57%)	0/35 (0%)	15/35 (43%)	0/35 (0%)
Role of JAK2 in Hormone-like Cytokine Signaling	0.001549	8.57	18/35 (51%)	0/35 (0%)	14/35 (40%)	3/35 (9%)
PI3K Signaling in B Lymphocytes	0.001585	3.91	66/128 (52%)	0/128 (0%)	57/128 (45%)	5/128 (4%)
Factors Promoting Cardiogenesis in Vertebrates	0.003236	4.35	52/92 (57%)	0/92 (0%)	37/92 (40%)	3/92 (3%)
TNFR1 Signaling	0.004169	6.12	27/49 (55%)	0/49 (0%)	20/49 (41%)	2/49 (4%)
**Antioxidant Action of Vitamin C**	0.004169	4.04	51/99 (52%)	0/99 (0%)	42/99 (42%)	6/99 (6%)
Acute Phase Response Signaling	0.005248	2.96	88/169 (52%)	0/169 (0%)	79/169 (47%)	2/169 (1%)
Differential Regulation of Cytokine Production in Macrophages and T Helper Cells by IL-17A and IL-17F	0.006166	11.1	7/18 (39%)	0/18 (0%)	11/18 (61%)	0/18 (0%)
**PPARα/RXRα Activation**	0.006607	2.79	98/179 (55%)	0/179 (0%)	67/179 (37%)	14/179 (8%)
Hepatic Fibrosis/Hepatic Stellate Cell Activation	0.007244	2.73	96/183 (52%)	0/183 (0%)	84/183 (46%)	3/183 (2%)
Type II Diabetes Mellitus Signaling	0.007586	3.42	50/117 (43%)	0/117 (0%)	64/117 (55%)	3/117 (3%)
Estrogen-Dependent Breast Cancer Signaling	0.008318	4.76	30/63 (48%)	0/63 (0%)	33/63 (52%)	0/63 (0%)
**LXR/RXR Activation**	0.008511	3.31	68/121 (56%)	0/121 (0%)	53/121 (44%)	0/121 (0%)
**RAR Activation**	0.008511	2.63	86/190 (45%)	0/190 (0%)	100/190 (53%)	4/190 (2%)

**Table 3:** Canonical pathways activated by 20,23(OH)_2_D3 in human epidermal keratinocytes after 6 h of treatment. Nuclear receptors are marked in bold.

Ingenuity Canonical Pathways	*p*-Value	Overlap (%)	Downregulated	No change	Upregulated	No Overlap with Dataset
2-ketoglutarate Dehydrogenase Complex	0.004898	25	2/4 (50%)	0/4 (0%)	2/4 (50%)	0/4 (0%)
**Aryl Hydrocarbon Receptor Signaling**	0.012589	1.43	64/140 (46%)	0/140 (0%)	70/140 (50%)	6/140 (4%)
Aldosterone Signaling in Epithelial Cells	0.014791	1.32	80/152 (53%)	0/152 (0%)	69/152 (45%)	3/152 (2%)
TCA Cycle II (Eukaryotic)	0.027542	4.35	14/23 (61%)	0/23 (0%)	9/23 (39%)	0/23 (0%)
Bupropion Degradation	0.0302	4	12/25 (48%)	0/25 (0%)	12/25 (48%)	1/25 (4%)
D-myo-inositol (1,4,5)-Trisphosphate Biosynthesis	0.032359	3.7	13/27 (48%)	0/27 (0%)	13/27 (48%)	1/27 (4%)
Acetone Degradation I (to Methylglyoxal)	0.032359	3.7	13/27 (48%)	0/27 (0%)	13/27 (48%)	1/27 (4%)
Xenobiotic Metabolism Signaling	0.042658	0.738	133/271 (49%)	0/271 (0%)	122/271 (45%)	16/271 (6%)
Estrogen Biosynthesis	0.045709	2.63	17/38 (45%)	0/38 (0%)	20/38 (53%)	1/38 (3%)
Nicotine Degradation III	0.064565	1.85	23/54 (43%)	0/54 (0%)	22/54 (41%)	9/54 (17%)
Melatonin Degradation I	0.067608	1.75	25/57 (44%)	0/57 (0%)	24/57 (42%)	8/57 (14%)
Superpathway of Melatonin Degradation	0.072444	1.61	27/62 (44%)	0/62 (0%)	27/62 (44%)	8/62 (13%)
GM-CSF Signaling	0.072444	1.61	26/62 (42%)	0/62 (0%)	36/62 (58%)	0/62 (0%)
Nicotine Degradation II	0.074131	1.59	26/63 (41%)	0/63 (0%)	25/63 (40%)	12/63 (19%)
**VDR/RXR Activation**	0.091201	1.28	34/78 (44%)	0/78 (0%)	43/78 (55%)	1/78 (1%)
Acute Myeloid Leukemia Signaling	0.091201	1.27	39/79 (49%)	0/79 (0%)	38/79 (48%)	2/79 (3%)
**TR/RXR Activation**	0.097724	1.18	39/85 (46%)	0/85 (0%)	46/85 (54%)	0/85 (0%)
Regulation of Actin-based Motility by Rho	0.105196	1.1	45/91 (49%)	0/91 (0%)	39/91 (43%)	7/91 (8%)
Antioxidant Action of Vitamin C	0.114025	1.01	53/99 (54%)	0/99 (0%)	40/99 (40%)	6/99 (6%)
Rac Signaling	0.119399	0.962	47/104 (45%)	0/104 (0%)	55/104 (53%)	2/104 (2%)
Type I Diabetes Mellitus Signaling	0.125893	0.909	54/110 (49%)	1/110 (1%)	46/110 (42%)	9/110 (8%)
RhoA Signaling	0.138676	0.82	56/122 (46%)	0/122 (0%)	61/122 (50%)	5/122 (4%)
3-phosphoinositide Biosynthesis	0.175792	0.633	72/158 (46%)	0/158 (0%)	76/158 (48%)	10/158 (6%)
RhoGDI Signaling	0.190985	0.578	75/173 (43%)	0/173 (0%)	94/173 (54%)	4/173 (2%)
Superpathway of Inositol Phosphate Compounds	0.212814	0.513	87/195 (45%)	0/195 (0%)	96/195 (49%)	12/195 (6%)
Actin Cytoskeleton Signaling	0.233884	0.461	93/217 (43%)	0/217 (0%)	115/217 (53%)	9/217 (4%)
Signaling by Rho Family GTPases	0.249459	0.427	106/234 (45%)	0/234 (0%)	124/234 (53%)	4/234 (2%)
Protein Ubiquitination Pathway	0.268534	0.392	121/255 (47%)	0/255 (0%)	126/255 (49%)	8/255 (3%)

**Table 4:** Ingenuity toxicity list secondary to keratinocytes treatment with 1,25(OH)_2_D3 in humans for 6 h. Nuclear receptors are marked in bold.

Ingenuity Toxicity Lists	*p*-Value	Overlap (%)	Downregulated	No Change	Upregulated	No Overlap with Dataset
**VDR/RXR Activation**	1.0 × 10^−10^	12.8	34/78 (44%)	0/78 (0%)	43/78 (55%)	1/78 (1%)
Hepatic Cholestasis	1.4 × 10^−5^	4.85	85/165 (52%)	0/165 (0%)	76/165 (46%)	4/165 (2%)
Renal Necrosis/Cell Death	2.8 × 10^−5^	2.59	241/501 (48%)	0/501 (0%)	242/501 (48%)	18/501 (4%)
**Aryl Hydrocarbon Receptor Signaling**	9.6 × 10^−5^	4.35	79/161 (49%)	0/161 (0%)	67/161 (42%)	15/161 (9%)
Liver Necrosis/Cell Death	0.000525	2.87	131/279 (47%)	0/279 (0%)	136/279 (49%)	12/279 (4%)
Liver Proliferation	0.000776	3.08	110/227 (48%)	0/227 (0%)	106/227 (47%)	11/227 (5%)
Cardiac Hypertrophy	0.00138	2.24	207/401 (52%)	0/401 (0%)	171/401 (43%)	23/401 (6%)
LPS/IL-1 Mediated Inhibition of RXR Function	0.00138	2.79	124/251 (49%)	0/251 (0%)	93/251 (37%)	34/251 (14%)
Hepatic Stellate Cell Activation	0.001549	8.57	17/35 (49%)	0/35 (0%)	18/35 (51%)	0/35 (0%)
Increases Liver Steatosis	0.002188	4.82	43/83 (52%)	0/83 (0%)	37/83 (45%)	3/83 (4%)
Mechanism of Gene Regulation by Peroxisome Proliferators via **PPARα**	0.003631	4.21	54/95 (57%)	0/95 (0%)	40/95 (42%)	1/95 (1%)
Hepatic Fibrosis	0.004169	4.04	42/99 (42%)	0/99 (0%)	54/99 (55%)	3/99 (3%)
Increases Liver Damage	0.005495	3.74	48/107 (45%)	0/107 (0%)	56/107 (52%)	3/107 (3%)
**PPARα/RXRα Activation**	0.007244	2.73	100/183 (55%)	0/183 (0%)	69/183 (38%)	14/183 (8%)
Acute Renal Failure Panel (Rat)	0.007943	4.84	31/62 (50%)	0/62 (0%)	24/62 (39%)	7/62 (11%)
**RAR Activation**	0.008511	2.63	86/190 (45%)	0/190 (0%)	100/190 (53%)	4/190 (2%)
Cardiac Necrosis/Cell Death	0.008913	2.23	141/269 (52%)	0/269 (0%)	114/269 (42%)	14/269 (5%)
Cardiac Fibrosis	0.008913	2.6	104/192 (54%)	0/192 (0%)	72/192 (38%)	16/192 (8%)
**LXR/RXR Activation**	0.008913	3.25	69/123 (56%)	0/123 (0%)	54/123 (44%)	0/123 (0%)
Nongenotoxic Hepatocarcinogenicity Biomarker Panel	0.00912	9.09	11/22 (50%)	0/22 (0%)	10/22 (45%)	1/22 (5%)
Increases Renal Damage	0.016218	3.7	38/81 (47%)	0/81 (0%)	36/81 (44%)	7/81 (9%)
NRF2-mediated Oxidative Stress Response	0.019498	2.14	99/234 (42%)	0/234 (0%)	103/234 (44%)	32/234 (14%)
TGF-β Signaling	0.021878	3.33	54/90 (60%)	0/90 (0%)	35/90 (39%)	1/90 (1%)

**Table 5:** Ingenuity toxicity list secondary to keratinocytes treatment with 20,23(OH)_2_D3 in humans for 6 h. Nuclear receptors are marked in bold.

Ingenuity Toxicity Lists	*p*-Value	Overlap (%)	Downregulated	No Change	Upregulated	No Overlap with Dataset
Cytochrome P450 Panel—Substrate is a Vitamin (Human)	0.007244	16.7	2/6 (33%)	0/6 (0%)	4/6 (67%)	0/6 (0%)
**Aryl Hydrocarbon Receptor Signaling**	0.016218	1.24	71/161 (44%)	0/161 (0%)	75/161 (47%)	15/161 (9%)
Cytochrome P450 Panel—Substrate is a Sterol (Human)	0.016982	7.14	5/14 (36%)	0/14 (0%)	9/14 (64%)	0/14 (0%)
Cytochrome P450 Panel—Substrate is a Xenobiotic (Human)	0.021878	5.56	7/18 (39%)	0/18 (0%)	9/18 (50%)	2/18 (11%)
Nongenotoxic Hepatocarcinogenicity Biomarker Panel	0.026303	4.55	12/22 (55%)	0/22 (0%)	9/22 (41%)	1/22 (5%)
Cytochrome P450 Panel—Substrate is a Xenobiotic (Mouse)	0.0302	4	5/25 (20%)	0/25 (0%)	7/25 (28%)	13/25 (52%)
Cytochrome P450 Panel—Substrate is a Xenobiotic (Rat)	0.030903	3.85	5/26 (19%)	0/26 (0%)	7/26 (27%)	14/26 (54%)
Xenobiotic Metabolism Signaling	0.063096	0.595	155/336 (46%)	0/336 (0%)	139/336 (41%)	42/336 (13%)
**VDR/RXR Activation**	0.091201	1.28	34/78 (44%)	0/78 (0%)	43/78 (55%)	1/78 (1%)
**TR/RXR Activation**	0.097724	1.18	39/85 (46%)	0/85 (0%)	46/85 (54%)	0/85 (0%)
Hepatic Fibrosis	0.114025	1.01	49/99 (49%)	0/99 (0%)	47/99 (47%)	3/99 (3%)
Increases Liver Hyperplasia/ Hyperproliferation	0.116145	0.99	39/101 (39%)	0/101 (0%)	52/101 (51%)	10/101 (10%)
Renal Necrosis/Cell Death	0.12388	0.399	224/501 (45%)	1/501 (0%)	258/501 (51%)	18/501 (4%)
Fatty Acid Metabolism	0.133352	0.855	57/117 (49%)	0/117 (0%)	39/117 (33%)	21/117 (18%)
Cardiac Fibrosis	0.209894	0.521	89/192 (46%)	0/192 (0%)	87/192 (45%)	16/192 (8%)
Liver Proliferation	0.24322	0.441	98/227 (43%)	0/227 (0%)	118/227 (52%)	11/227 (5%)

**Table 6:** Categories of biological functions with diseases or function annotation activated by 1,25(OH)_2_D3 in human epidermal keratinocytes after 6 h of treatment.

Categories of Biological Function	Diseases or Functions Annotation	*p*-Value	Activation z-Score	# of Genes
Cancer, Organismal Injury and Abnormalities	growth of tumor	1.5 × 10^−12^	0.033	31
Cellular Movement	cell movement	4.5 × 10^−12^	1.845	52
Cancer, Cellular Development, Cellular Growth and Proliferation, Organismal Injury and Abnormalities, Tumor Morphology	proliferation of tumor cells	7.5 × 10^−12^	−0.27	23
Cellular Movement	migration of cells	1.3 × 10^−11^	1.801	48
Cellular Growth and Proliferation	proliferation of cells	4.2 × 10^−11^	0.347	70
Carbohydrate Metabolism	metabolism of polysaccharide	4.4 × 10^−11^	0.755	16
Cell Death and Survival	apoptosis of tumor cell lines	8.3 × 10^−11^	1.089	36
Cellular Movement	invasion of cells	8.7 × 10^−11^	0.571	30
Inflammatory Response	inflammatory response	1.4 × 10^−10^	0.766	28
Cellular Development	differentiation of cells	1.5 × 10^−10^	2.774	51
Cell Death and Survival	cell survival	3.1 × 10^−10^	2.346	38
Cell Death and Survival	apoptosis	5.7 × 10^−10^	1.307	55
Cell Death and Survival	necrosis	6.3 × 10^−10^	1.7	54
Cell Death and Survival	cell viability	6.4 × 10^−10^	2.547	36
Tissue Morphology	quantity of cells	7.1 × 10^−10^	1.065	43
Cardiovascular System Development and Function, Organismal Development	vasculogenesis	9.4 × 10^−10^	0.844	26
Carbohydrate Metabolism	synthesis of polysaccharide	9.6 × 10^−10^	0.297	13
Cellular Growth and Proliferation, Tissue Development	proliferation of connective tissue cells	1.2 × 10^−9^	1.232	23
Cellular Movement	cell movement of tumor cell lines	1.2 × 10^−9^	0.955	28
Cell Death and Survival	cell death of tumor cell lines	1.2 × 10^−9^	0.81	39
Cellular Development, Cellular Growth and Proliferation	proliferation of tumor cell lines	1.9 × 10^−9^	0.324	39
Organismal Survival	morbidity or mortality	2.3 × 10^−9^	−1.006	51
Embryonic Development, Organismal Development	development of body trunk	2.7 × 10^−9^	0.06	32
Cell Death and Survival	cell death	3.1 × 10^−9^	1.556	62
Cancer, Organismal Injury and Abnormalities	growth of malignant tumor	3.3 × 10^−9^	0.518	19
Cellular Development, Cellular Growth and Proliferation, Connective Tissue Development and Function, Tissue Development	proliferation of fibroblasts	4.0 × 10^−9^	0.604	17
Cellular Function and Maintenance, Hematological System Development and Function	function of myeloid cells	4.5 × 10^−9^		14
Cell Signaling, Small Molecule Biochemistry	synthesis of nitric oxide	4.7 × 10^−9^	0.89	15
Dermatological Diseases and Conditions	psoriasis	5.0 × 10^−9^		22

**Table 7:** Categories of biological functions with diseases or function annotation activated by 20,23(OH)_2_D3 in human epidermal keratinocytes after 6 h of treatment.

Categories of Biological Function	Diseases or Functions Annotation	*p*-Value	# of Genes
Cancer, Organismal Injury and Abnormalities, Respiratory Disease	carcinoma in lung	6.5 × 10^−5^	8
Vitamin and Mineral Metabolism	metabolism of vitamin	0.000254	3
Developmental Disorder, Skeletal and Muscular Disorders	hypertrophy of smooth muscle	0.000325	2
Cancer, Organismal Injury and Abnormalities, Respiratory Disease	non-small cell lung cancer	0.000334	7
Infectious Diseases	internalization of virus	0.000355	2
Cancer, Organismal Injury and Abnormalities	adenocarcinoma	0.000603	20
Cancer, Organismal Injury and Abnormalities, Reproductive System Disease	prostate cancer	0.000618	7
Cancer, Gastrointestinal Disease, Hepatic System Disease, Organismal Injury and Abnormalities	liver adenoma	0.000692	2
Cancer, Endocrine System Disorders, Organismal Injury and Abnormalities	endocrine gland tumor	0.000718	9
Cell Death and Survival	apoptosis of germ cells	0.000799	3
Lipid Metabolism, Small Molecule Biochemistry, Vitamin and Mineral Metabolism	catabolism of terpenoid	0.000828	2
Cancer, Organismal Injury and Abnormalities	epithelial cancer	0.000989	23
Endocrine System Development and Function, Lipid Metabolism, Small Molecule Biochemistry, Vitamin and Mineral Metabolism	synthesis of estrogen	0.00114	2
Ophthalmic Disease, Organismal Injury and Abnormalities	age-related macular degeneration type 6	0.00122	1
Cell Cycle	arrest in sub-G1 phase of endometrial cancer cell lines	0.00122	1
Cell Morphology, Connective Tissue Development and Function	blebbing of pulmonary fibroblasts	0.00122	1
Organismal Injury and Abnormalities	calcification of uterus	0.00122	1
Cell Cycle, Cell Death and Survival	chromosome condensation of pulmonary fibroblasts	0.00122	1
Cell-To-Cell Signaling and Interaction, Inflammatory Response	cytotoxic reaction of bone marrow cells	0.00122	1
Tissue Morphology	deficiency of mast cells	0.00122	1
Cell Cycle	delay in G1/S phase transition of hepatoma cell lines	0.00122	1
Embryonic Development, Organ Development, Organismal Development, Tissue Development, Visual System Development and Function	development of outflow pathway	0.00122	1
Embryonic Development, Organ Development, Organismal Development, Reproductive System Development and Function, Tissue Development	development of placenta decidua	0.00122	1
Embryonic Development, Organ Development, Organismal Development, Reproductive System Development and Function, Tissue Development	development of placental spongiotrophoblast layer	0.00122	1
Cardiovascular System Development and Function, Tissue Morphology	diameter of portal vein	0.00122	1
Cardiovascular System Development and Function, Tissue Morphology	diameter of umbilical vein	0.00122	1
Hereditary Disorder, Ophthalmic Disease, Organismal Injury and Abnormalities	digenic early-onset glaucoma	0.00122	1
Cancer, Organismal Injury and Abnormalities, Reproductive System Disease	estrogen receptor positive endometrial cancer	0.00122	1
Connective Tissue Disorders, Organismal Injury and Abnormalities	fibrosis of submucosa	0.00122	1
Digestive System Development and Function, Embryonic Development, Organ Development, Organismal Development, Tissue Development	formation of salivary duct	0.00122	1
Cardiovascular System Development and Function, Embryonic Development, Lymphoid Tissue Structure and Development, Organ Development, Organismal Development, Respiratory System Development and Function, Tissue Development	formation of tracheal duct	0.00122	1
Cancer, Gastrointestinal Disease, Organismal Injury and Abnormalities	hyperplasia of pylorus	0.00122	1
Cancer, Cardiovascular Disease, Organismal Injury and Abnormalities	hyperplasia of vasculature	0.00122	1
Developmental Disorder, Gastrointestinal Disease	hypertrophy of gastric epithelium	0.00122	1
Dermatological Diseases and Conditions, Developmental Disorder	hypertrophy of skin	0.00122	1

**Table 8:** Canonical pathways activated by 1,25(OH)_2_D3 in human epidermal keratinocytes after 24 h of treatment. Nuclear receptors are marked in bold.

Ingenuity Canonical Pathways	*p*-Value	Overlap (%)	Downregulated	No Change	Upregulated	No Overlap with Dataset
**VDR/RXR Activation**	7.9 × 10^−15^	15.4	23/78 (29%)	0/78 (0%)	54/78 (69%)	1/78 (1%)
MIF-mediated Glucocorticoid Regulation	2.6 × 10^−5^	12.1	12/33 (36%)	0/33 (0%)	20/33 (61%)	1/33 (3%)
MIF Regulation of Innate Immunity	6.3 × 10^−5^	9.76	19/41 (46%)	0/41 (0%)	21/41 (51%)	1/41 (2%)
α-tocopherol Degradation	0.000162181	50	0/4 (0%)	0/4 (0%)	4/4 (100%)	0/4 (0%)
Antioxidant Action of Vitamin C	0.000177828	5.05	43/99 (43%)	0/99 (0%)	50/99 (51%)	6/99 (6%)
Retinoate Biosynthesis I	0.000691831	9.09	9/33 (27%)	0/33 (0%)	20/33 (61%)	4/33 (12%)
Coagulation System	0.000831764	8.57	17/35 (49%)	0/35 (0%)	18/35 (51%)	0/35 (0%)
Estrogen Biosynthesis	0.001047129	7.89	18/38 (47%)	0/38 (0%)	19/38 (50%)	1/38 (3%)
iNOS Signaling	0.00162181	6.82	17/44 (39%)	0/44 (0%)	26/44 (59%)	1/44 (2%)
Role of IL-17A in Arthritis	0.002884032	5.56	27/54 (50%)	0/54 (0%)	27/54 (50%)	0/54 (0%)
Parkinson’s Signaling	0.003162278	12.5	9/16 (56%)	0/16 (0%)	7/16 (44%)	0/16 (0%)
**LXR/RXR Activation**	0.003890451	3.31	61/121 (50%)	0/121 (0%)	60/121 (50%)	0/121 (0%)
CD40 Signaling	0.004897788	4.62	31/65 (48%)	0/65 (0%)	33/65 (51%)	1/65 (2%)
IL-10 Signaling	0.005495409	4.41	32/68 (47%)	0/68 (0%)	35/68 (51%)	1/68 (1%)
Role of MAPK Signaling in the Pathogenesis of Influenza	0.005754399	4.35	27/69 (39%)	0/69 (0%)	39/69 (57%)	3/69 (4%)
LPS/IL-1 Mediated Inhibition of RXR Function	0.006025596	2.28	102/219 (47%)	0/219 (0%)	105/219 (48%)	12/219 (5%)
Role of Osteoblasts, Osteoclasts and Chondrocytes in Rheumatoid Arthritis	0.006025596	2.28	111/219 (51%)	0/219 (0%)	103/219 (47%)	5/219 (2%)
IL-17 Signaling	0.006456542	4.17	33/72 (46%)	0/72 (0%)	39/72 (54%)	0/72 (0%)
LPS-stimulated MAPK Signaling	0.00676083	4.11	33/73 (45%)	0/73 (0%)	40/73 (55%)	0/73 (0%)
Toll-like Receptor Signaling	0.007079458	4.05	32/74 (43%)	0/74 (0%)	40/74 (54%)	2/74 (3%)
BMP signaling pathway	0.007585776	3.95	43/76 (57%)	0/76 (0%)	31/76 (41%)	2/76 (3%)
Intrinsic Prothrombin Activation Pathway	0.01023293	6.9	12/29 (41%)	0/29 (0%)	16/29 (55%)	1/29 (3%)
4-1BB Signaling in T Lymphocytes	0.011481536	6.45	19/31 (61%)	0/31 (0%)	12/31 (39%)	0/31 (0%)
Acute Phase Response Signaling	0.012302688	2.37	76/169 (45%)	0/169 (0%)	91/169 (54%)	2/169 (1%)
Endothelin-1 Signaling	0.013182567	2.33	89/172 (52%)	0/172 (0%)	77/172 (45%)	6/172 (3%)
Inhibition of Angiogenesis by TSP1	0.013803843	5.88	21/34 (62%)	0/34 (0%)	11/34 (32%)	2/34 (6%)
Xenobiotic Metabolism Signaling	0.014454398	1.85	127/271 (47%)	0/271 (0%)	128/271 (47%)	16/271 (6%)
IL-17A Signaling in Fibroblasts	0.014454398	5.71	15/35 (43%)	0/35 (0%)	20/35 (57%)	0/35 (0%)
Interferon Signaling	0.015488166	5.56	28/36 (78%)	0/36 (0%)	8/36 (22%)	0/36 (0%)
Thyroid Hormone Biosynthesis	0.015848932	33.3	1/3 (33%)	0/3 (0%)	2/3 (67%)	0/3 (0%)
April Mediated Signaling	0.016982437	5.26	18/38 (47%)	0/38 (0%)	20/38 (53%)	0/38 (0%)
Inhibition of Matrix Metalloproteases	0.017782794	5.13	17/39 (44%)	0/39 (0%)	21/39 (54%)	1/39 (3%)
**RAR Activation**	0.018197009	2.11	87/190 (46%)	0/190 (0%)	99/190 (52%)	4/190 (2%)
Thrombin Signaling	0.018620871	2.09	90/191 (47%)	0/191 (0%)	96/191 (50%)	5/191 (3%)
B Cell Activating Factor Signaling	0.018620871	5	21/40 (53%)	0/40 (0%)	19/40 (48%)	0/40 (0%)
Dermatan Sulfate Biosynthesis (Late Stages)	0.022387211	4.55	16/44 (36%)	0/44 (0%)	26/44 (59%)	2/44 (5%)
IL-6 Signaling	0.023442288	2.59	52/116 (45%)	0/116 (0%)	64/116 (55%)	0/116 (0%)
p38 MAPK Signaling	0.023988329	2.56	52/117 (44%)	0/117 (0%)	62/117 (53%)	3/117 (3%)

**Table 9:** Canonical pathways activated by 20,23(OH)_2_D3 in human epidermal keratinocytes after 24 h of treatment. Nuclear receptors are marked in bold.

Ingenuity Canonical Pathways	*p*-Value	Overlap (%)	Downregulated	No Change	Upregulated	No Overlap with Dataset
**Aryl Hydrocarbon Receptor Signaling**	9.5 × 10^−10^	22.9	80/140 (57%)	0/140 (0%)	54/140 (39%)	6/140 (4%)
Superpathway of Cholesterol Biosynthesis	9.1 × 10^−9^	46.4	22/28 (79%)	0/28 (0%)	4/28 (14%)	2/28 (7%)
Cell Cycle Control of Chromosomal Replication	6.3 × 10^−8^	44.4	21/27 (78%)	0/27 (0%)	6/27 (22%)	0/27 (0%)
Mismatch Repair in Eukaryotes	2.3 × 10^−7^	56.2	15/16 (94%)	0/16 (0%)	1/16 (6%)	0/16 (0%)
Unfolded protein response	3.5 × 10^−7^	29.6	24/54 (44%)	0/54 (0%)	29/54 (54%)	1/54 (2%)
Fatty Acid α-oxidation	1.5 × 10^−6^	47.4	10/19 (53%)	0/19 (0%)	6/19 (32%)	3/19 (16%)
Ethanol Degradation IV	6.8 × 10^−6^	40.9	17/22 (77%)	0/22 (0%)	2/22 (9%)	3/22 (14%)
Cholesterol Biosynthesis I	7.9 × 10^−6^	53.8	12/13 (92%)	0/13 (0%)	1/13 (8%)	0/13 (0%)
p53 Signaling	8.3 × 10^−6^	20.4	53/98 (54%)	0/98 (0%)	45/98 (46%)	0/98 (0%)
**VDR/RXR Activation**	1.5 × 10^−5^	21.8	26/78 (33%)	0/78 (0%)	51/78 (65%)	1/78 (1%)
GADD45 Signaling	1.7 × 10^−5^	42.1	14/19 (74%)	0/19 (0%)	5/19 (26%)	0/19 (0%)
Putrescine Degradation III	2.8 × 10^−5^	40	12/20 (60%)	0/20 (0%)	5/20 (25%)	3/20 (15%)
Histamine Degradation	4.5 × 10^−5^	43.8	10/16 (63%)	0/16 (0%)	3/16 (19%)	3/16 (19%)
Dopamine Degradation	4.7 × 10^−5^	33.3	16/27 (59%)	0/27 (0%)	6/27 (22%)	5/27 (19%)
Tryptophan Degradation X (Mammalian, via Tryptamine)	6.2 × 10^−5^	36.4	13/22 (59%)	0/22 (0%)	5/22 (23%)	4/22 (18%)
Xenobiotic Metabolism Signaling	9.3 × 10^−5^	13.3	131/271 (48%)	0/271 (0%)	124/271 (46%)	16/271 (6%)
Oxidative Ethanol Degradation III	0.000109648	38.9	13/18 (72%)	0/18 (0%)	2/18 (11%)	3/18 (17%)
Mevalonate Pathway I	0.000112202	46.2	8/13 (62%)	0/13 (0%)	3/13 (23%)	2/13 (15%)
Estrogen-mediated S-phase Entry	0.000125893	33.3	18/24 (75%)	0/24 (0%)	6/24 (25%)	0/24 (0%)
Hereditary Breast Cancer Signaling	0.000165959	16.3	77/129 (60%)	0/129 (0%)	45/129 (35%)	7/129 (5%)
**Glucocorticoid Receptor Signaling**	0.000269153	12.7	122/275 (44%)	0/275 (0%)	147/275 (53%)	6/275 (2%)
Adipogenesis pathway	0.000371535	15.7	66/127 (52%)	0/127 (0%)	55/127 (43%)	6/127 (5%)
Interferon Signaling	0.000537032	25	24/36 (67%)	0/36 (0%)	12/36 (33%)	0/36 (0%)
Superpathway of Serine and Glycine Biosynthesis I	0.000630957	57.1	3/7 (43%)	0/7 (0%)	4/7 (57%)	0/7 (0%)
Superpathway of Geranylgeranyldiphosphate Biosynthesis I (via Mevalonate)	0.000630957	35.3	11/17 (65%)	0/17 (0%)	4/17 (24%)	2/17 (12%)
Semaphorin Signaling in Neurons	0.000758578	20.8	27/53 (51%)	0/53 (0%)	24/53 (45%)	2/53 (4%)
Glutaryl-CoA Degradation	0.000776247	41.7	7/12 (58%)	0/12 (0%)	4/12 (33%)	1/12 (8%)
Pancreatic Adenocarcinoma Signaling	0.000794328	16	53/106 (50%)	0/106 (0%)	53/106 (50%)	0/106 (0%)
Role of CHK Proteins in Cell Cycle Checkpoint Control	0.001047129	20	35/55 (64%)	0/55 (0%)	20/55 (36%)	0/55 (0%)
Glycolysis I	0.001096478	28	20/25 (80%)	0/25 (0%)	4/25 (16%)	1/25 (4%)
NRF2-mediated Oxidative Stress Response	0.001230269	13.3	89/180 (49%)	0/180 (0%)	87/180 (48%)	4/180 (2%)
HIF1α Signaling	0.001412538	15.7	52/102 (51%)	0/102 (0%)	48/102 (47%)	2/102 (2%)
Aldosterone Signaling in Epithelial Cells	0.001548817	13.8	82/152 (54%)	0/152 (0%)	67/152 (44%)	3/152 (2%)
Serotonin Degradation	0.001737801	17.9	38/67 (57%)	0/67 (0%)	16/67 (24%)	13/67 (19%)

**Table 10:** Toxicity-related pathways identified by Ingenuity in human keratinocytes treated with 1,25(OH)_2_D3 for 24 h. Nuclear receptors are marked in bold.

Ingenuity Toxicity Lists	*p*-Value	Overlap (%)	Downregulated	No Change	Upregulated	No Overlap with Dataset
**VDR/RXR Activation**	7.9 × 10^−15^	15.4	23/78 (29%)	0/78 (0%)	54/78 (69%)	1/78 (1%)
Xenobiotic Metabolism Signaling	0.00040738	2.38	144/336 (43%)	0/336 (0%)	150/336 (45%)	42/336 (13%)
Cardiac Fibrosis	0.000537032	3.12	85/192 (44%)	0/192 (0%)	91/192 (47%)	16/192 (8%)
Cytochrome P450 Panel—Substrate is an Eicosanoid (Human)	0.000562341	28.6	1/7 (14%)	0/7 (0%)	6/7 (86%)	0/7 (0%)
Cytochrome P450 Panel—Substrate is a Fatty Acid (Human)	0.001202264	20	3/10 (30%)	0/10 (0%)	7/10 (70%)	0/10 (0%)
Cardiac Hypertrophy	0.00128825	2	193/401 (48%)	0/401 (0%)	185/401 (46%)	23/401 (6%)
Liver Proliferation	0.00128825	2.64	103/227 (45%)	0/227 (0%)	113/227 (50%)	11/227 (5%)
Hepatic Fibrosis	0.001862087	4.04	49/99 (49%)	1/99 (1%)	46/99 (46%)	3/99 (3%)
**LXR/RXR Activation**	0.004073803	3.25	62/123 (50%)	0/123 (0%)	61/123 (50%)	0/123 (0%)
Renal Necrosis/Cell Death	0.005011872	1.6	238/501 (48%)	0/501 (0%)	245/501 (49%)	18/501 (4%)
LPS/IL-1 Mediated Inhibition of RXR Function	0.010715193	1.99	107/251 (43%)	0/251 (0%)	110/251 (44%)	34/251 (14%)
Positive Acute Phase Response Proteins	0.010964782	6.67	11/30 (37%)	0/30 (0%)	19/30 (63%)	0/30 (0%)
Liver Necrosis/Cell Death	0.016218101	1.79	118/279 (42%)	0/279 (0%)	149/279 (53%)	12/279 (4%)
**RAR Activation**	0.018197009	2.11	87/190 (46%)	0/190 (0%)	99/190 (52%)	4/190 (2%)
Increases Liver Damage	0.019054607	2.8	46/107 (43%)	0/107 (0%)	58/107 (54%)	3/107 (3%)
Fatty Acid Metabolism	0.023988329	2.56	48/117 (41%)	0/117 (0%)	48/117 (41%)	21/117 (18%)
Increases Liver Hepatitis	0.030902954	3.85	22/52 (42%)	0/52 (0%)	29/52 (56%)	1/52 (2%)

**Table 11:** Toxicity-related pathways identified by Ingenuity in human keratinocytes treated with 20,23(OH)_2_D3 for 24 h. Nuclear receptors are marked in bold.

Ingenuity Toxicity Lists	*p*-Value	Overlap (%)	Downregulated	No Change	Upregulated	No Overlap with Dataset
**Aryl Hydrocarbon Receptor Signaling**	2.6 × 10^−9^	21.1	90/161 (56%)	0/161 (0%)	56/161 (35%)	15/161 (9%)
Cholesterol Biosynthesis	1.1 × 10^−8^	62.5	13/16 (81%)	0/16 (0%)	3/16 (19%)	0/16 (0%)
Renal Necrosis/Cell Death	1.9 × 10^−7^	13.2	228/501 (46%)	0/501 (0%)	255/501 (51%)	18/501 (4%)
Primary Glomerulonephritis Biomarker Panel (Human)	1.7 × 10^−6^	63.6	5/11 (45%)	0/11 (0%)	6/11 (55%)	0/11 (0%)
p53 Signaling	2.6 × 10^−6^	21.2	54/99 (55%)	0/99 (0%)	45/99 (45%)	0/99 (0%)
**VDR/RXR Activation**	1.5 × 10^−5^	21.8	26/78 (33%)	0/78 (0%)	51/78 (65%)	1/78 (1%)
Liver Proliferation	3.0 × 10^−5^	14.5	96/227 (42%)	0/227 (0%)	120/227 (53%)	11/227 (5%)
Cardiac Hypertrophy	5.1 × 10^−5^	12.2	176/401 (44%)	0/401 (0%)	202/401 (50%)	23/401 (6%)
Liver Necrosis/Cell Death	7.8 × 10^−5^	13.3	117/279 (42%)	0/279 (0%)	150/279 (54%)	12/279 (4%)
Oxidative Stress	0.000380189	21.1	39/57 (68%)	0/57 (0%)	17/57 (30%)	1/57 (2%)
**Mechanism of Gene Regulation by Peroxisome Proliferators via PPARα**	0.000645654	16.8	38/95 (40%)	0/95 (0%)	56/95 (59%)	1/95 (1%)
Xenobiotic Metabolism Signaling	0.000794328	11.6	152/336 (45%)	0/336 (0%)	142/336 (42%)	42/336 (13%)
Increases Renal Proliferation	0.002398833	13.9	68/137 (50%)	0/137 (0%)	62/137 (45%)	7/137 (5%)
Fatty Acid Metabolism	0.002398833	14.5	65/117 (56%)	0/117 (0%)	31/117 (26%)	21/117 (18%)
Increases Liver Steatosis	0.003890451	15.7	32/83 (39%)	0/83 (0%)	48/83 (58%)	3/83 (4%)
Decreases Depolarization of Mitochondria and Mitochondrial Membrane	0.004570882	25	18/24 (75%)	0/24 (0%)	5/24 (21%)	1/24 (4%)
Cell Cycle: G1/S Checkpoint Regulation	0.004677351	16.7	35/66 (53%)	0/66 (0%)	28/66 (42%)	3/66 (5%)

**Table 12:** Categories of biological functions with diseases or function annotation activated by 1,25(OH)_2_D3 in human epidermal keratinocytes after 24 of treatment.

Categories of Biological Function	Diseases or Functions Annotation	*p*-Value	Activation z-Score	# of Genes
Cancer, Organismal Injury and Abnormalities	benign neoplasia	1.0 × 10^−9^	0.927	26
Dermatological Diseases and Conditions	psoriasis	2.6 × 10^−9^		20
Cardiovascular System Development and Function, Cellular Movement	cell movement of endothelial cells	8.8 × 10^−9^	1.37	15
Cancer, Cellular Movement, Organismal Injury and Abnormalities, Tumor Morphology	invasion of tumor cells	1.2 × 10^−8^	1.596	11
Cell Signaling, Small Molecule Biochemistry	synthesis of nitric oxide	1.9 × 10^−8^	−0.217	13
Cardiovascular System Development and Function, Cellular Movement	homing of endothelial cells	3.2 × 10^−8^	1.597	7
Cancer, Organismal Injury and Abnormalities, Tumor Morphology	invasion of tumor	3.4 × 10^−8^	1.63	12
Lipid Metabolism, Small Molecule Biochemistry	metabolism of eicosanoid	3.4 × 10^−8^	2.747	12
Cardiovascular Disease, Hematological Disease	Thrombosis	5.9 × 10^−8^	−0.946	10
Organismal Injury and Abnormalities	Fibrosis	1.1 × 10^−7^	−1.401	17
Lipid Metabolism, Small Molecule Biochemistry	metabolism of prostaglandin	1.2 × 10^−7^	2.589	10
Immunological Disease	hypersensitive reaction	1.5 × 10^−7^	0.914	15
Cardiovascular System Development and Function, Organismal Development	vasculogenesis	1.6 × 10^−7^	1.825	20
Inflammatory Response	inflammation of organ	1.8 × 10^−7^	−0.022	26
Dermatological Diseases and Conditions, Inflammatory Disease, Inflammatory Response	Dermatitis	1.8 × 10^−7^	−0.355	15
Cardiovascular System Development and Function, Organismal Development	vascularization of hindlimb	2.3 X 10^−7^	1.994	4
Cellular Movement	homing	3.4 × 10^−7^	1.468	17
Cancer, Cellular Development, Cellular Growth and Proliferation, Organismal Injury and Abnormalities, Tumor Morphology	proliferation of tumor cells	5.4 × 10^−7^	−0.189	15
Cancer, Organismal Injury and Abnormalities	growth of tumor	6.0 × 10^−7^	0.402	20
Cellular Movement	invasion of cells	6.3 × 10^−7^	1.731	21

**Table 13:** Categories of biological functions with diseases or function annotation activated by 20,23(OH)_2_D3 in human epidermal keratinocytes after 24 h of treatment.

Categories of Biological Function	Diseases or Functions Annotation	*p*-Value	Activation z-Score	# of Genes
Cellular Growth and Proliferation	proliferation of cells	7.0 × 10^−33^	−3.052	551
Dermatological Diseases and Conditions	psoriasis	4.6 × 10^−29^		142
Cell Death and Survival	cell death	1.2 × 10^−27^	1.116	493
Cell Death and Survival	necrosis	1.9 × 10^−26^	1.15	400
Cell Death and Survival	apoptosis	1.9 × 10^−25^	0.619	405
Cell Death and Survival	cell death of tumor cell lines	7.2 × 10^−25^	0.971	266
Cell Death and Survival	apoptosis of tumor cell lines	7.6 × 10^−23^	0.672	219
Cellular Movement	cell movement	1.3 × 10^−22^	−0.187	334
Cellular Movement	migration of cells	6.7 × 10^−21^	−0.554	301
Cancer, Organismal Injury and Abnormalities	abdominal neoplasm	8.7 × 10^−20^	−1.733	1030
Infectious Diseases	Viral Infection	1.2 × 10^−19^	0.737	261
Cancer, Organismal Injury and Abnormalities	tumorigenesis of tissue	6.5 × 10^−19^	−0.349	1047
Cancer, Organismal Injury and Abnormalities	abdominal cancer	2.3 × 10^−18^	−1.938	1014
Cancer, Organismal Injury and Abnormalities	cancer	4.2 × 10^−18^	1.528	1215
Cancer, Organismal Injury and Abnormalities	neoplasia of epithelial tissue	2.3 × 10^−17^	−0.365	1026
Cellular Development, Cellular Growth and Proliferation	proliferation of tumor cell lines	3.0 × 10^−17^	−2.431	245
Cancer, Organismal Injury and Abnormalities	benign neoplasia	3.5 × 10^−17^	−0.029	165
Cell Death and Survival	cell survival	4.7 × 10^−17^	−0.537	225
Cancer, Organismal Injury and Abnormalities	advanced stage solid tumor	7.1 × 10^−17^	−0.397	106

**Table 14:** Glide XP scores of vitamin D3 analogs docked into the refined human AhR LBD model.

Compound	Score	Compound	Score
20*S*OHD3	−13.3	1,20*S*,23*S*(OH)_3_D3	−16.1
1,25(OH)_2_D3	−13.1	1,20*S*,23*R*(OH)_3_D3	−16.4
20*S*,23*S*(OH)_2_D3	−15.1	17,20*S*,23*S*(OH)_3_D3	−14.9
20*S*,23*R*(OH)_2_D3	−15.7	17,20*S*,23*R*(OH)_3_D3	−15.4
1,20*S*(OH)_2_D3	−13.0		

## Supplementary Materials

Supplementary materials can be found at http://www.mdpi.com/1422-0067/19/10/3072/s1.

## Abbreviations

1,20(OH)_2_D3	1,20-dihyhydroxyvitamin D3
17,20,23(OH)_2_D3	17,20,23-trihyhydroxyvitamin D3
1α,25(OH)_2_D3	1α,25-dihydroxyvitamin D3
20(OH)D3	20-hydroxyvitamin D3
20,23(OH)_2_D3	20,23-dihyhydroxyvitamin D3
25(OH)D3	25-hydroxyvitamin D3
7-DHC	7-dehydrocholesterol
AA	African-American
AhR	aryl hydrocarbon receptor
GR	glucocorticoid receptor
LBD	ligand binding domain
LXR	liver X receptor
PPAR	peroxisome proliferator-activated receptor
RAR	retinoic acid receptor
RXR	retinoid X receptor
VDR	vitamin D receptor
UVB	ultraviolet B radiation
