# Supplementary material for: Differential and Overlapping Effects of 20,23(OH)2D3 and 1,25(OH)2D3 on Gene Expression in Human Epidermal Keratinocytes: Identification of AhR as an Alternative Receptor for 20,23(OH)2D3
Source: Int J Mol Sci. 2018 Oct 8;19(10):3072. doi: 10.3390/ijms19103072 (PMC6213311; doi:10.3390/ijms19103072)
Supplement: Supplementary file 1 [file ijms-19-03072-s001.zip › Supplemental-figures-and-table.doc]

**Supplemental figures and table**

**Supplemental figure 1**. VDR/RXR signaling pathway activated by 1,25(OH)_2_D3 in neonatal epidermal keratinocytes from AA after 6 (**A**) and 24 h (**B**) of treatment. Relative gene expression is marked red (stimulation) and green (inhibition).

**Supplemental figure 2**. AhR signaling pathway activated by 20,23(OH)_2_D3 in in neonatal epidermal keratinocytes after 6 (**A**) and 24 h (**B**) of treatment. Relative gene expression is marked red (stimulation) and green (inhibition).

**Supplemental figure 3**. VDR/RXR signaling pathway activated by 20,23(OH)_2_D3 in in neonatal epidermal keratinocytes after 6 (**A**) and 24 h (**B**) of treatment. Relative gene expression is marked red (stimulation) and green (inhibition).

**Supplemental figure 4.** Sequence alignment used for homology modelling the ligand binding domain of the human AhR sequence (Uniprot ID P35869).

**Supplemental figure 5.** Ligand - protein contacts are shown versus simulation time.

**Supplemental figure 6.** Induced Fit docked Vitamin D3 analogs displayed simultaneously. AhR residues in proximity of docked ligands are shown.

**Supplemental table 1.** Sequences of primers

| Primer | left | right |
| --- | --- | --- |
| Cyclophilin B | TGTGGTGTTTGGCAAAGTTC | GTTTATCCCGGCTGTCTGTC |
| GAPDH | AGCCACATCGCTCAGACAC | GCCCAATACGACCAAATCCC |
| CYP24 | CATCATGGCCATCAAAACAAT | GCAGCTCGACTGGAGTGAC |
| CYP1A1 | CGGCCCCGGCTCTCT | GTGTCGGAAGGTCTCCCAGGAT |
| CYP1B1 | ATATGACTGGAGCCGACTTTCC | GGCGAACTTTATCGGGTTGA |
| AhR | CAG TTA CCT CCG GGT GAA GA | CCA GCG CAA AGC CAT TAA GA |
